# B cell overexpression of FCRL5 and PD-1 is associated with low antibody titers in HCV infection

**DOI:** 10.1371/journal.ppat.1010179

**Published:** 2022-01-06

**Authors:** Clinton O. Ogega, Nicole E. Skinner, Andrew I. Flyak, Kaitlyn E. Clark, Nathan L. Board, Pamela J. Bjorkman, James E. Crowe, Andrea L. Cox, Stuart C. Ray, Justin R. Bailey

**Affiliations:** 1 Division of Infectious Diseases, Department of Medicine, Johns Hopkins University School of Medicine; Baltimore, Maryland, United States of America; 2 Division of Biology and Biological Engineering, California Institute of Technology; Pasadena, California, United States of America; 3 Department of Pathology, Microbiology and Immunology, Vanderbilt University Medical Center; Nashville, Tennessee, United States of America; 4 Department of Pediatrics, Vanderbilt University Medical Center; Nashville, Tennessee, United States of America; 5 Vanderbilt Vaccine Center, Vanderbilt University Medical Center; Nashville, Tennessee, United States of America; Institut Pasteur, FRANCE

## Abstract

Antibodies targeting the hepatitis C virus (HCV) envelope glycoprotein E2 are associated with delayed disease progression, and these antibodies can also facilitate spontaneous clearance of infection in some individuals. However, many infected people demonstrate low titer and delayed anti-E2 antibody responses. Since a goal of HCV vaccine development is induction of high titers of anti-E2 antibodies, it is important to define the mechanisms underlying these suboptimal antibody responses. By staining lymphocytes with a cocktail of soluble E2 (sE2) glycoproteins, we detected HCV E2-specific (sE2+) B cells directly *ex vivo* at multiple acute infection timepoints in 29 HCV-infected subjects with a wide range of anti-E2 IgG titers, including 17 persistently infected subjects and 12 subjects with spontaneous clearance of infection. We performed multi-dimensional flow cytometric analysis of sE2+ and E2-nonspecific (sE2-) class-switched B cells (csBC). In sE2+ csBC from both persistence and clearance subjects, frequencies of resting memory B cells (rMBC) were reduced, frequencies of activated MBC (actMBC) and tissue-like MBC (tlMBC) were increased, and expression of FCRL5, an IgG receptor, was significantly upregulated. Across all subjects, plasma anti-E2 IgG levels were positively correlated with frequencies of sE2+ rMBC and sE2+ actMBC, while anti-E2 IgG levels were negatively correlated with levels of FCRL5 expression on sE2+ rMBC and PD-1 expression on sE2+ actMBC. Upregulation of FCRL5 on sE2+ rMBC and upregulation of PD-1 on sE2+ actMBC may limit anti-E2 antibody production *in vivo*. Strategies that limit upregulation of these molecules could potentially generate higher titers of protective antibodies against HCV or other pathogens.

## Author summary

Antiviral immunity relies on production of protective immunoglobulin G (IgG) by B cells, but many hepatitis C virus (HCV)-infected individuals have very low levels of HCV-specific IgG in their serum. Elucidating mechanisms underlying this suboptimal IgG expression remains paramount in guiding therapeutic and vaccine strategies. In this study, we developed a highly specific method to capture HCV-specific B cells and characterized their surface protein expression. Two proteins analyzed were Fc receptor-like protein 5 (FCRL5), a cell surface receptor for IgG, and programmed cell death protein-1 (PD-1), a marker of lymphocyte activation and exhaustion. We measured serum levels of anti-HCV IgG in these subjects and demonstrated that overexpression of FCRL5 and PD-1 on memory B cells was associated with reduced anti-E2 IgG levels. This study uses HCV as a viral model, but the findings may be applicable to many viral infections, and they offer new potential targets to enhance antiviral IgG production.

## Introduction

More than 71 million people worldwide are chronically infected with hepatitis C virus (HCV), and persistent infection can lead to liver failure and hepatocellular carcinoma [1–3]. There are approximately 1.75 million new HCV infections annually, and from 2000 to 2016, the number of deaths attributed to HIV, malaria, and tuberculosis declined while those associated with HCV increased [4, 5]. Despite the high efficacy of direct-acting antivirals (DAAs) for HCV treatment, few countries are on pace to achieve the goal set by the World Health Organization to eliminate HCV as a public health problem by 2030 [6]. Therefore, elimination probably will require the development of an effective prophylactic HCV vaccine.

Investigation of the immune responses of persons infected with HCV can inform vaccine development. Neutralizing antibodies targeting the envelope glycoprotein E2 are associated with delayed disease progression in chronically infected individuals [7, 8], but many chronically infected individuals demonstrate delayed or low-titer anti-E2 antibody responses. Spontaneous clearance of infection, which occurs in about 25% of infected individuals, has been associated with early development of IgG directed against E2 [9] and early development of E2-specific broadly neutralizing antibodies (bNAbs), which are capable of blocking infection by diverse HCV isolates [10–14]. However, like those with persistent infection, some individuals with spontaneous clearance demonstrate low-titer anti-E2 antibody responses [15–20]. In these cases, clearance may be predominantly T cell-mediated [21–23]. Since a goal of HCV vaccine development is induction of high titers of anti-E2 antibodies, it is important to define the mechanisms underlying suboptimal antibody responses in natural infection.

We hypothesized that B cell dysfunction contributes to poor anti-E2 responses in some individuals. The recent development of methods for flow cytometric staining of E2-specific B cells using soluble E2 (sE2) proteins as bait has finally allowed detection of the antigen-specific cells responsible for the anti-E2 antibody response [24, 25], but phenotypic evaluation of these cells has thus far been limited. B cells’ functional status can be evaluated by quantitation of subsets, including resting memory B cells (rMBC, CD21high, CD27high), activated MBC (actMBC, CD21low, CD27high), intermediate MBC (intMBC, CD21high, CD27low), and tissue-like MBC (tlMBC, CD21low, CD27low). In addition, B cell function can also be evaluated by measuring cell surface expression of inhibitory receptors including Siglec 2 (CD22) [26, 27], B and T lymphocyte attenuator (BTLA) [28], and programmed cell death protein-1 (PD-1) [29, 30]. Some studies of chronic viral infections have also identified abnormal B cell expression of IgG receptors (including FCRL5) [31, 32], and chemokine receptors (including CXCR5) [33].

To identify the mechanisms underlying suboptimal anti-E2 antibody responses in HCV infection, we measured the frequencies and cell-surface molecule expression profiles of E2-specific B cells isolated at longitudinal acute infection timepoints from HCV-infected individuals with a wide range of plasma anti-E2 IgG titers, including individuals with either persistence or spontaneous clearance of infection. To facilitate these analyses, we first developed and optimized a sensitive and highly specific protocol for staining of E2-specific B cells, using a cocktail of three rationally-selected, antigenically-distinct sE2 proteins. We then used this protocol to quantitate HCV E2-specific (sE2+) class-switched B cells (csBC) at multiple acute infection timepoints. We measured the frequencies of sE2+ csBC subsets, including intMBC, rMBC, actMBC, and tlMBC. To further characterize sE2+ B cells, we measured cell-surface expression of BTLA, CD22, PD-1, FCRL5, and CXCR5. Finally, we analyzed correlations between sE2+ csBC subset frequencies, surface molecule expression, and plasma anti-E2 IgG levels.

## Results

### Enhanced sensitivity of E2-specific B cell detection using a sE2 cocktail

**Fig 1:**
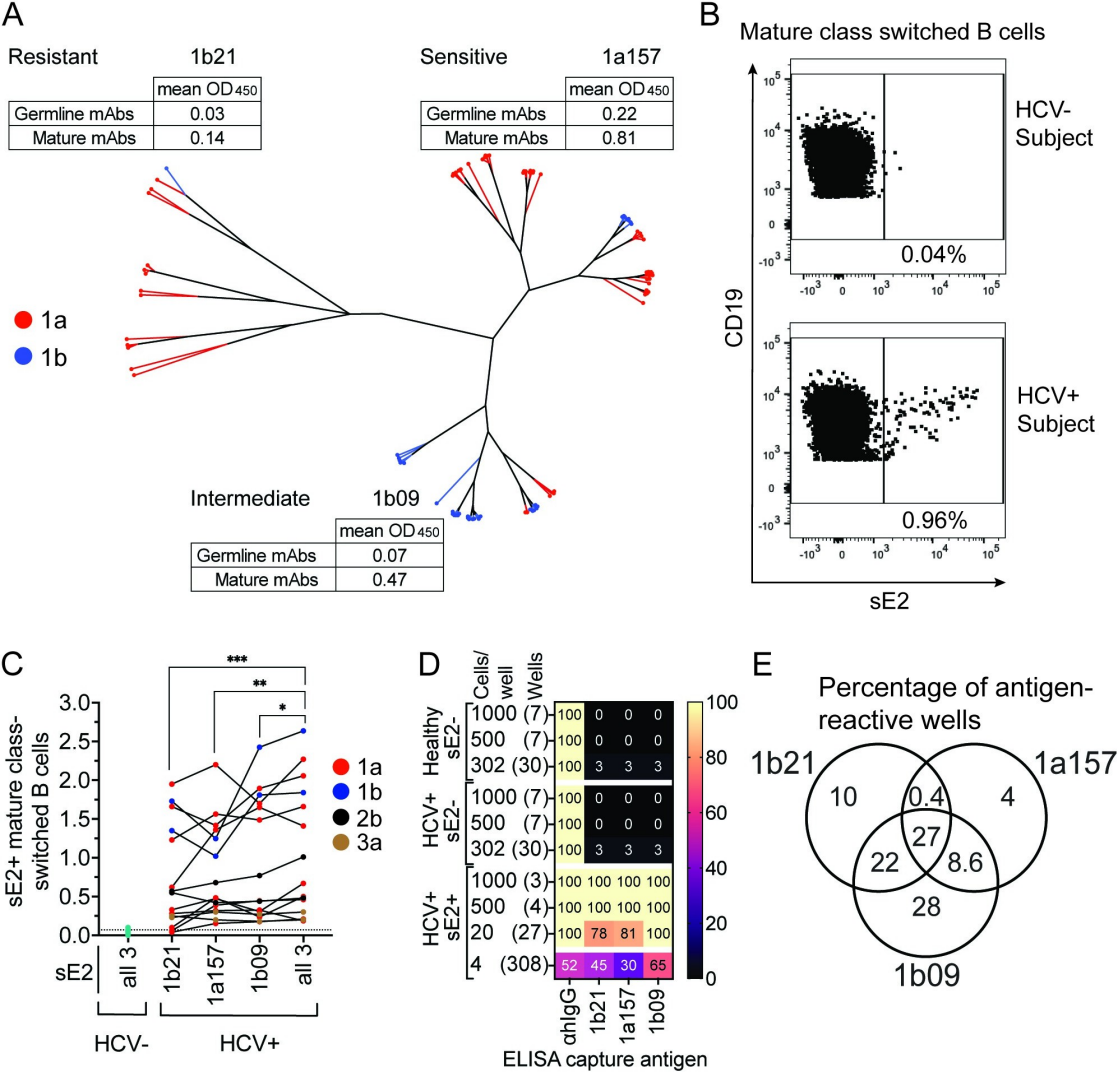
Enhanced sensitivity of E2-specific B cell detection using a sE2 cocktail. **(A)** Hierarchical clustering of 89 genetically distinct sE2 proteins based upon relative binding of 12 E2-specific and 12 inferred reverted unmutated ancestor (rua) mAbs. Red lines indicate subtype 1a, and blue lines indicate subtype 1b proteins. Mean binding (OD450nm) of mature or rua mAbs to sE2s representative of each cluster is shown. **(B)** Representative staining of sE2-specific class switched B cells (csBC; CD3-, CD19+, IgM-, IgD-, CD10-, sE2+) in a healthy (HCV-) or an HCV+ subject. **(C)** Frequency (%) of csBC that are sE2+ in 15 HCV+ subjects, using either single sE2 proteins or a cocktail of three proteins. Dotted line indicates the true-positive threshold set using staining of cells from healthy, HCV- subjects. **(D)** Percentage of cultured csBC well supernatants that produced IgG or reacted in an ELISA with the sE2 antigens. **(E)** Percentage of E2-specific supernatants from 4 B cells/well cultures that reacted to each sE2 used in the cocktail. In **C**, non-normally distributed data by the Shapiro Wilk normality test was compared using the matched Friedman test with p values adjusted for multiple comparisons using the Two-stage linear step-up procedure of Benjamini, Krieger and Yekutieli. * P ≤ 0.05, ** P ≤ 0.01, *** P ≤ 0.001.

To identify optimal bait antigens for staining of E2-specific B cells, we subcloned E2 genes from a previously described diverse set of genotype 1a and 1b E1E2 clones encoding functional glycoproteins [34], generating a novel set of 89 soluble E2 (sE2) expression constructs. To express soluble proteins, E2 genes were truncated as previously described [35] to delete the transmembrane domain and add a C-terminal 6X-Histidine tag. To confirm native folding and to define antigenic characteristics of these sE2 proteins, we used an ELISA to measure binding of each sE2 by a panel of 12 previously described anti-E2 human monoclonal antibodies (mAbs) [36, 37], as well as 12 inferred germline precursors of these mAbs, which were generated by site-directed mutagenesis of mature mAb variable sequences (HEPC74, HEPC3, AR3C, AR3A, HEPC85, HEPC98, HEPC108, HEPC132, HEPC146, HEPC151, HEPC153, and HEPC154; S1 Fig). This set of reference mAbs was selected to represent the wide variety of anti-E2 antibodies that arise *in vivo*. The mAb panel includes both broadly neutralizing and weakly neutralizing antibodies, targeting a variety of conformational epitopes across multiple E2 domains (HVR1, E2 front layer, CD81 binding loop, central beta sandwich, and back layer). Germline mAb precursors were included to represent antibodies that have not undergone affinity maturation. We performed a hierarchical clustering analysis based on sE2-mAb ELISA results, grouping sE2s based on their patterns of relative sensitivity to binding across the panel of 24 mAbs (Fig 1A). The 89 sE2 proteins segregated into three major clusters of antigenically distinct proteins. We chose one representative sE2 protein from each cluster (clones designated 1a157 (subtype 1a), 1b09 (subtype 1b), and 1b21 (subtype 1b)) to test individually and in combination as bait antigens. In addition to their distinct patterns of relative sensitivity across the mAb panel, we also noted that of the three proteins, 1a157 was most sensitive to mAb binding, 1b21 was most resistant, and 1b09 showed intermediate sensitivity.

To test these sE2 bait antigens, we stained PBMCs obtained a median of 345 days post infection (dpi) (range, 80–2,370) from fifteen subjects infected with HCV subtypes 1a, 1b, 2b, or 3a. PBMCs from each subject were stained with 1a157, 1b09, or 1b21 sE2s individually, or with a cocktail of all three proteins at the same total protein concentration. Cells were stained simultaneously with antibodies specific for classical B-cell markers to identify mature, class-switched B cells (csBC) (CD19+, CD3-, CD10-, IgM-, IgD-) (S2 Fig). PBMCs from healthy, HCV negative subjects were included in all experiments to determine the frequency of nonspecifically sE2-stained cells (Fig 1B and 1C). We observed varying frequencies of sE2 specific (sE2+) csBC across different subjects (range 0.2 to 2.6% using the sE2 cocktail) (Fig 1C). The frequencies across all subjects were significantly higher using the cocktail of three sE2 proteins together relative to staining with 1b21, 1a157, or 1b09 individually (p = 0.0007, 0.007, or 0.02, respectively) (Fig 1C). From these data, we concluded that a rationally-selected cocktail of sE2s was more sensitive than single sE2 proteins for detection of circulating E2-specific B cells.

To confirm the specificity of the sE2 staining protocol, we stained 20 million PBMCs from a subtype 1b HCV-infected subject and a healthy, HCV-negative donor with the sE2 cocktail and antibodies against CD19, CD3, CD10, IgM, and IgD (S3A Fig). We sorted sE2+ csBC from the HCV+ subject, and sE2- csBC from both the HCV+ subject and the healthy donor. We cultured these sorted csBC at densities of 1,000, 500, 302, 20, or 4 B cells per well in a 96-well plate format. The csBC were stimulated with recombinant IL-2, IL-21 and irradiated CD40L-expressing feeder cells to induce their differentiation into antibody-secreting plasmablasts [38, 39]. We performed ELISAs with culture supernatants from each well to measure production of total IgG as well as IgG specific for each of the three bait sE2 antigens (Figs 1D and S3B–S3E).

All wells cultured at densities of 20 or greater B cells per well, and 52% of wells cultured at a density of 4 B cells per well, were positive for IgG production. None of the wells (0/14) containing sE2- B cells cultured at densities of 1,000 and 500 cells per well were E2 reactive. Only 2 of 60 wells (3%) containing sE2- B cells cultured at density of 302 B cells/well were E2 reactive (one well each from the HCV+ subject and the healthy donor). In contrast, all sE2+ B cell wells cultured at 1,000 or 500 B cells/well reacted with all three sE2 antigens. sE2+ wells cultured at 20 B cells/well reacted with 1a157, 1b09, and 1b21 sE2 at frequencies of 22/27 wells (81%), 27/27 wells (100%), and 21/27 wells (78%), respectively. Of wells cultured with 4 sE2+ B cells, 93/308 wells (30%) reacted with 1a157, 199/308 (65%) reacted with 1b09, and 138/308 (45%) reacted with 1b21. Notably, among all E2 reactive wells cultured at 4 B cells/well, 9 (4%) reacted only with 1a157, 65 (28%) reacted only with 1b09, and 23 (10%) reacted only with 1b21 (Figs 1E and S3B–S3E). Taken together, these data demonstrate that the sE2 staining protocol is highly specific and can be used to detect B cells that recognize diverse E2 variants. Furthermore, the data demonstrate the benefit of staining with a cocktail of antigenically diverse sE2 antigens, since between 14% and 38% of E2-reactive B cells in this experiment would not have been detected by staining with a single sE2 antigen.

### Quantitation of HCV sE2 specific csBC

After the sensitivity and specificity of our staining protocol was established, we used it to measure frequencies and cell surface molecule expression of sE2+ and sE2- csBC from 17 subjects who were transitioning to persistent HCV infection (Persistence group), and from 12 subjects who spontaneously cleared HCV infection without treatment (Clearance group) (Table 1). Samples were acquired from the prospective, longitudinal Baltimore Before and After Acute Study of Hepatitis (BBAASH) cohort, allowing us to study samples acquired during acute infection. We studied two timepoints from each subject: an early and later timepoint in Persistence subjects and a pre- and post-clearance timepoint in Clearance subjects (Table 1). Pre-Clearance samples were selected to fall as near as possible to the last viremic timepoint prior to clearance of infection. Twelve of the Persistence subjects were selected based on availability of samples that were time-matched for days post infection (dpi) with Clearance subjects’ pre- and post-Clearance samples. HCV viral loads did not differ significantly between pre-Clearance samples and time matched Persistence samples, or between early and late Persistence samples (Table 1).

**Table 1:** Subject characteristics.

	Clearance (n = 12)		Persistence (n = 17)				
	Pre-Cle.^1^	Post-Cle.^1^	Per. TM-Pre.^1^^,^^2^	Per. TM-Post^1^^,^^3^	Early Per.^1^	Late Per.^1^	P-value^4^
dpi^5^ median (range)	89.75 (21–644)	163.25 (78–821)	97.75 (62–642)	162.75 (106–1023)	101 (57–156)	352 (170–642)	0.6, >0.9
**Genotype**							
1a	5		7	8	9	9	
1b	2		0	2	1	1	
2b	1		4	2	2	2	
3a	4		1	0	1	0	
RNA IU mean (range)	1.5e6 (25–10.8e6)	<20 (0-<20)	2.4e6 (244–8.1e6)	2.6e6 (5,620–7.5e6)	3e6 (181–8.1e6)	1.6e6 (439–7.8e6)	0.2, <0.0001
Analyzed sE2+ csBC median (range)	76 (19–1012)	79 (10–1227)	102 (34–599)	193 (52–959)	102 (34–686)	220 (37–1083)	

^1^Cle. = Clearance, Per. = Persistence, TM-Pre = Time Matched to Pre-Clearance samples, TM-Post = Time matched to Post-Clearance samples.

^2^11 of 12 and

^3^9 of 12 time-matched (TM) Per. subjects overlap with Early or Late Per. (n = 13).

^4^Pre-Cle. vs Per. TM-Pre or Post-Cle. vs Per. TM-Post, respectively. Unpaired two-tailed Mann-Whitney U Test for non-normally distributed data by Shapiro-Wilk test was used.

^5^days post-infection.

**Fig 2:**
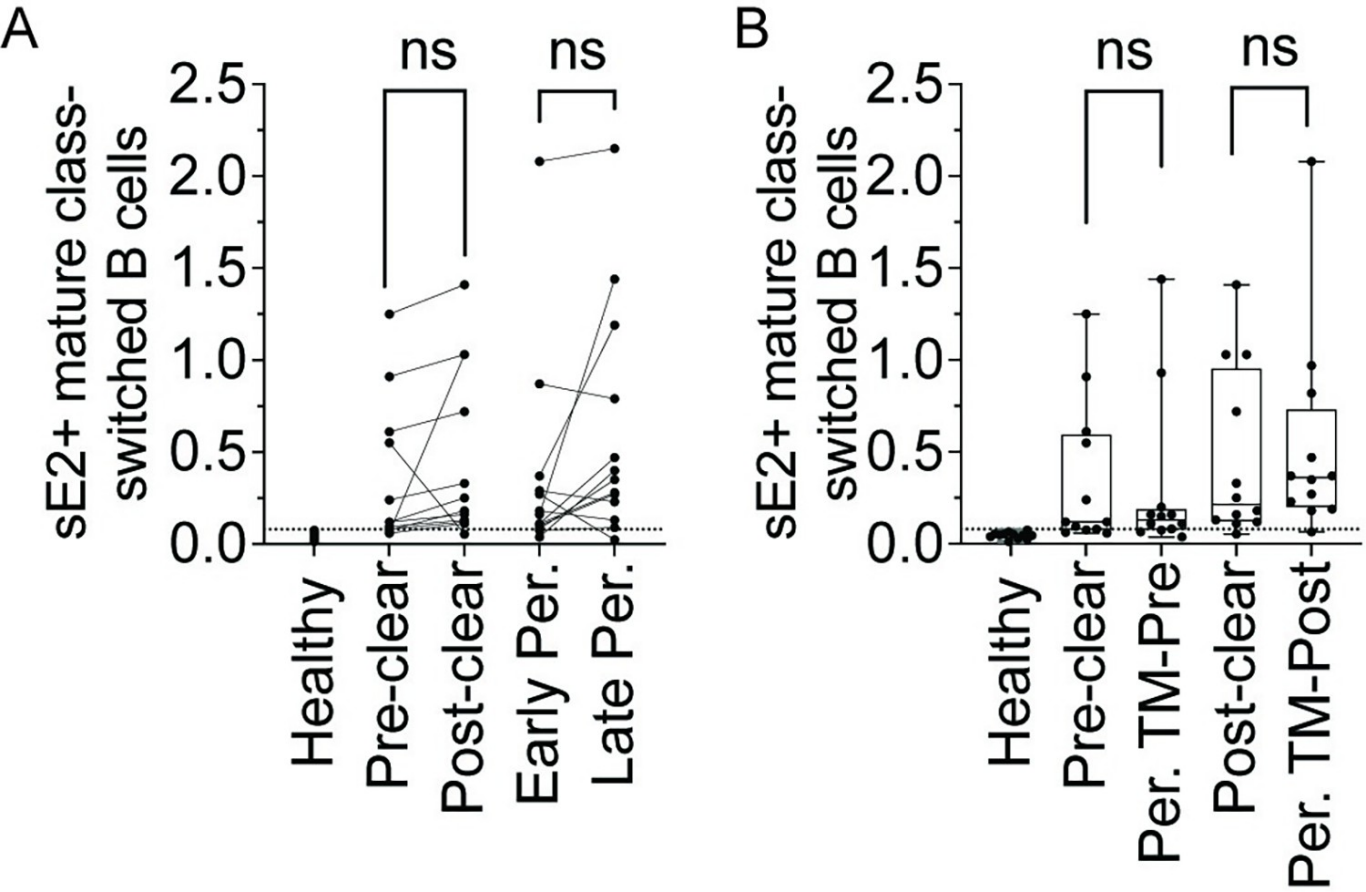
Equivalent frequencies of sE2-specific B cells in Clearance or Persistence subjects. **(A)** Frequency (%) of class switched B cells that are sE2-specific (sE2+ csBC; CD3-, CD19+, IgM-, IgD-, CD10-, sE2+) at two timepoints from 12 Clearance or 13 Persistence subjects. Lines link samples from the same subject. **(B)** Comparison of % sE2+ csBC between Clearance subjects (pre-Clear, post-Clear) or Persistence subjects time-matched with pre- or post-Clearance samples for duration of infection (Per. TM-Pre, Per. TM-Post). Dotted line represents the true-positive threshold, defined as the mean frequency plus two standard deviations in the healthy group. Each point indicates a single sample. In **B**, Horizontal lines indicate means, boxes are inter-quartile range, and whiskers are minimum to maximum. Normality of data was determined using Shapiro Wilk normality test. Comparisons in **A** were performed using paired two-tailed Student’s T test and in **B**, using unpaired Mann-Whitney U test with p values adjusted using the Bonferroni method. ns = not significant.

We quantitated the frequencies of sE2+ cells among csBC from early and later Persistence samples and pre- and post-Clearance samples. Eleven of thirteen (84.6%) early and twelve of thirteen (92.3%) later Persistence timepoints had a detectable frequency of sE2+ mature csBC above the true positive threshold, which was set based upon staining in HCV- healthy controls. Two Persistence subjects who were negative for sE2+ csBC at the early timepoint were positive at the later timepoint, while one subject with low positive frequency at the early timepoint became negative at the later timepoint (Fig 2A). Nine of twelve (75%) pre-Clearance and eleven of twelve (92%) post-Clearance samples had a frequency of sE2+ csBC above the true positive threshold. Three Clearance subjects who were negative for sE2+ csBC at the pre-Clearance timepoint were positive at the post-Clearance timepoint, while one subject with low positive frequency at the pre-Clearance timepoint became negative at the later timepoint (Fig 2A). There were no significant differences in the frequencies of sE2+ csBC from Clearance subjects’ pre- or post-clearance samples and their time-matched Persistence samples (Fig 2B). Overall, these data demonstrate that most Persistence and Clearance subjects have detectable sE2+ csBC in circulation at both early acute and later infection timepoints.

### Expansion of sE2+ actMBC and tissue-like memory B cell subsets

Next, we compared cell-surface molecule expression on sE2+ and sE2- csBC. To do this, we used conventional manual gating as well as geometric mean fluorescence intensities (MFI) to measure the cell surface expression of CD21, CD27, BTLA, CD22, CXCR5, FCRL5, and PD-1. For analyses of sE2+ B cell subsets and cell surface molecule expression, we analyzed 43 and excluded 7 samples with sE2+ csBC frequencies below the true-positive cutoff.

**Fig 3:**
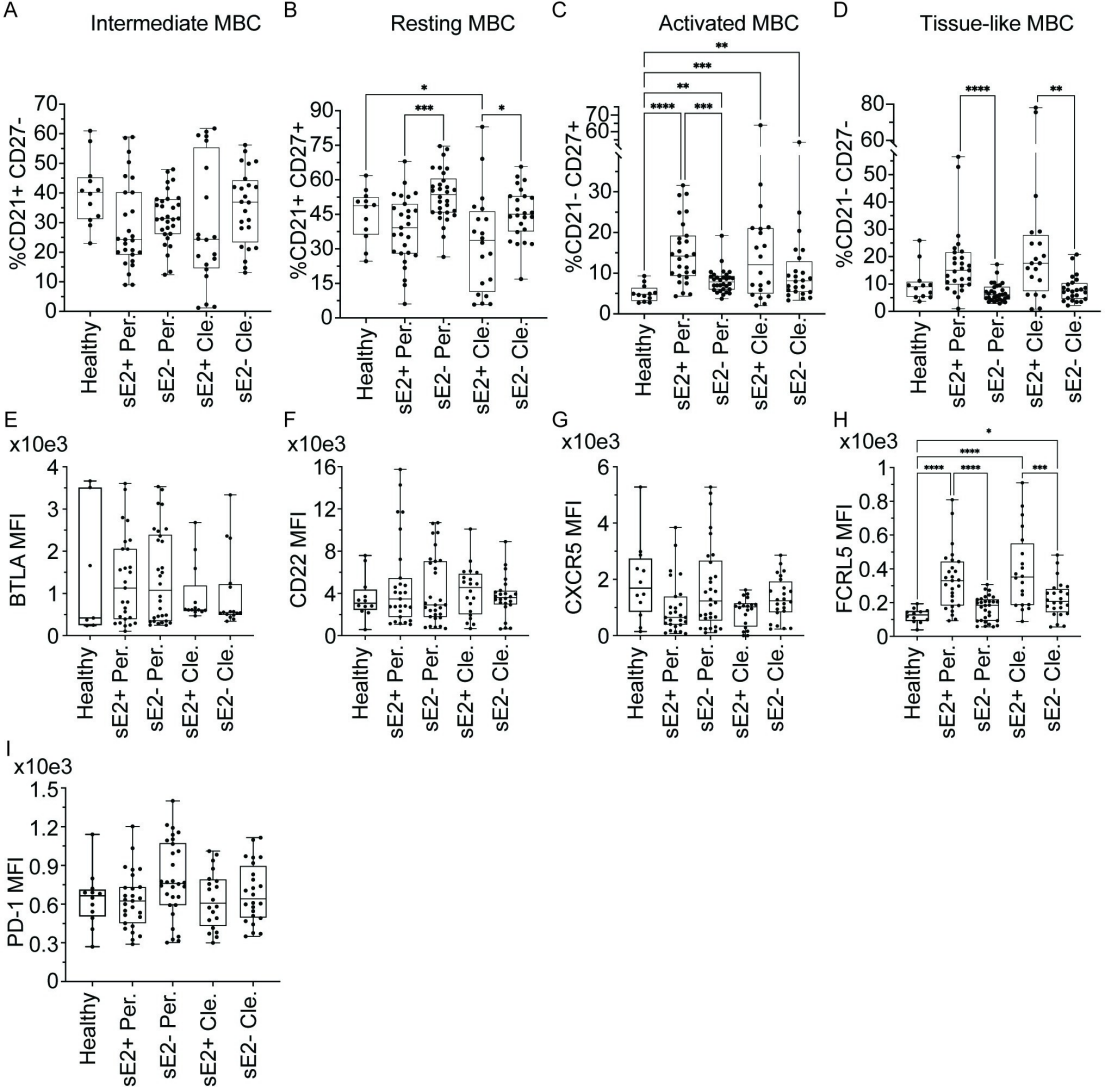
Expansion of actMBC and tlMBC, with upregulation of FCRL5 expression on sE2+ csBC. For **A-D**, frequency (%) of **(A)** intermediate MBC (intMBC, CD21high, CD27low), **(B)** resting MBC (rMBC, CD21high, CD27high), **(C)** activated MBC (actMBC, CD21low, CD27high), or **(D)** tissue-like memory B cells (tlMBC, CD21low, CD27low) subsets. For **E-I**, geometric mean fluorescence intensity (MFI) of (**E**) BTLA, (**F**) CD22, (**G**) FCRL5, (**H**) CXCR5, or (**I**) PD-1. Samples compared are sE2-nonspecific (sE2-) or sE2-specific (sE2+) csBC from healthy, Persistence (Per.), or Clearance (Cle.) subjects, with each point indicating a single sample. Early Persistence, late Persistence, pre-Clearance, and post-Clearance samples are included. Horizontal lines indicate means, boxes are inter-quartile range, and whiskers are minimum to maximum. Normality of data was determined using the Shapiro Wilk normality test. Comparisons were performed using the Kruskal-Wallis test for non-normally distributed data (**A, C-D, E-F, H**) or one-way ANOVA for normally distributed data (**B, G, I**), with p values adjusted for multiple comparisons using the Benjamini, Krieger and Yekutieli method. Only statistically significant comparisons are indicated (* P ≤ 0.05, ** P ≤ 0.01, *** P ≤ 0.001, **** P ≤ 0.0001).

CD21 and CD27 expression on mature, csBC allows for the identification of intermediate MBC (intMBC, CD21high, CD27low), resting MBC (rMBC, CD21high, CD27high), activated MBC (actMBC, CD21low, CD27high), or tissue-like memory B cells (tlMBC, CD21low, CD27low) (S2 Fig). In all comparisons between healthy, Persistence, and Clearance samples, we did not observe any differences in the frequencies of intMBC between sE2+ and sE2- csBC (Fig 3A). In Persistence samples, frequencies of rMBC among sE2+ csBC were reduced compared to frequencies among sE2- csBC (p = 0.0009), while in Clearance samples, frequencies of rMBC among sE2+ csBC were decreased relative to frequencies among sE2- csBC from the same subjects or healthy donors (p = 0.04 and 0.01, respectively) (Fig 3B). Frequencies of actMBC were significantly increased among all sE2+ and sE2- csBC from Persistence and Clearance samples relative to samples from healthy controls (Fig 3C). In Persistence samples, actMBC also were significantly more frequent among sE2+ csBC than among sE2- csBC (p = 0.0009) (Fig 3C). In both Persistence and Clearance samples, tlMBC were more frequent among sE2+ csBC than among sE2- cells (p<0.0001, Persistence; p = 0.004, Clearance) (Fig 3D).

To investigate any differences between time-matched samples from Clearance and Persistence subjects, we also compared the frequency of B cell subsets in sE2+ csBC from pre-Clearance samples and time-matched Persistence samples. We did not detect significant differences in the frequencies of sE2+ intMBC, rMBC, actMBC, or tlMBC cells between these groups (S4A Fig). We also did not detect significant differences in the frequencies of these sE2+ B cell subsets in early versus late Persistence samples or pre- versus post-Clearance samples (S4B and S4C Fig). Taken together, these data demonstrate that in both Persistence and Clearance samples, frequencies of rMBC are reduced and frequencies of actMBC and tlMBC cells are expanded in sE2+ csBC relative to sE2- csBC.

### Upregulation of FCRL5 and PD-1 expression on sE2+ csBC subsets

We further expanded our analysis by evaluating differences in cell surface expression of inhibitory molecules (FCRL5, CD22, BTLA, PD-1) and a chemokine receptor (CXCR5) on sE2+ and sE2- csBC. We did not see significant differences in the geometric mean fluorescence intensity (MFI) of BTLA, CD22, CXCR5 or PD-1 expression on sE2+ or sE2- csBC from Persistence, Clearance, or healthy control subjects (Fig 3E–3G and 3I). However, for both Persistence and Clearance samples, FCRL5 expression was significantly greater on sE2+ csBC relative to sE2- csBC from the same subjects or healthy controls (p = <0.0001 for all comparisons) (Fig 3H). There were no statistically significant findings in the comparisons of any of the surface molecules on sE2+ csBC between pre-Clearance and time-matched Persistence samples, between early and late Persistence samples, or between pre- and post-Clearance samples (S5 Fig).

**Fig 4:**
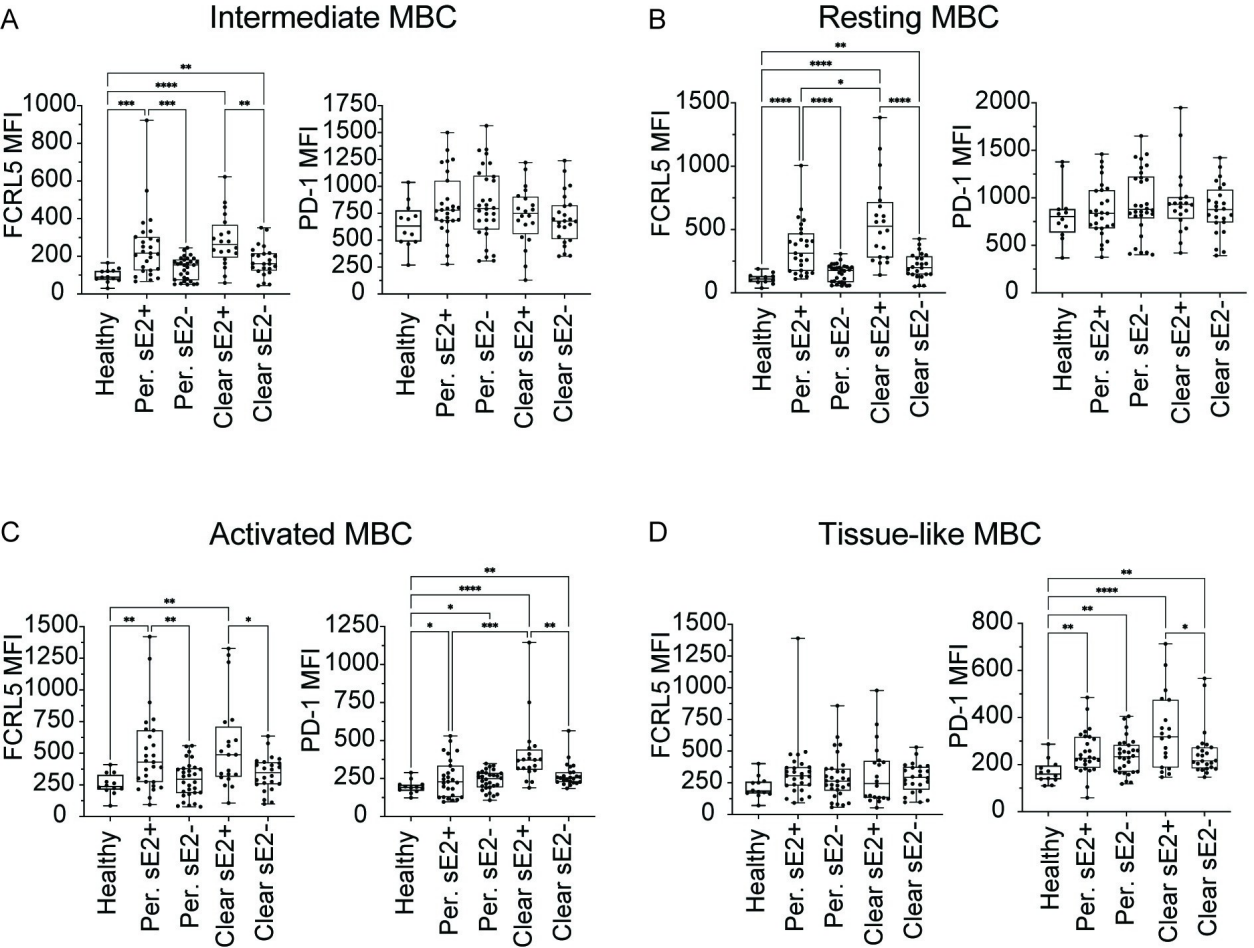
Upregulation of FCRL5 and PD-1 expression on sE2+ csBC subsets. Surface expression of FCRL5 or PD-1 on sE2-nonspecific (sE2-) or sE2-specific (sE2+) (**A**) intermediate MBC (intMBC, CD21high, CD27low), (**B**) resting MBC (rMBC, CD21high, CD27high), (**C**) activated MBC (actMBC, CD21low, CD27high), or (**D**) tissue-like memory B cells (tlMBC, CD21low, CD27low) from healthy, Persistence, or Clearance subjects. Each point indicates a single sample. Early Persistence, late Persistence, pre-Clearance, and post-Clearance samples are included. Geometric mean fluorescence intensity (MFI) is used for both receptors. Horizontal lines indicate means, boxes are inter-quartile range, and whiskers are minimum to maximum. Normality of data was determined using Shapiro Wilk normality test. Comparisons in **A-D** were performed using the Kruskal-Wallis test for non-normally distributed data (A-D), with p values adjusted for multiple comparisons using the Benjamini, Krieger and Yekutieli method. Only statistically significant comparisons are indicated (* P ≤ 0.05, ** P ≤ 0.01, *** P ≤ 0.001, **** P ≤ 0.0001).

Having observed increased expression of FCRL5 on sE2+ csBC, we next evaluated surface molecule expression on csBC subsets (intMBC, rMBC, actMBC, and tlMBC). In both Persistence and Clearance samples, FCRL5 was significantly upregulated on sE2+ cells relative to cells from healthy donors on intMBC (p = 0.0001, <0.0001), rMBC (p = <0.0001, <0.0001), and actMBC (p = 0.005, 0.004) subsets, but not on tlMBC (p = 0.4, 0.4). FCRL5 expression similarly was increased in both Persistence and Clearance samples on sE2+ cells relative to sE2- cells from the same donors on intMBC (p = 0.0009, 0.004), rMBC (p = <0.0001, 0.0001), and actMBC (p = 0.005, 0.05) subsets, but not on tlMBC (p = 0.5, 0.7) (Fig 4A–4D). Moreover, FCRL5 expression on sE2+ rMBC was somewhat higher in Clearance samples than in Persistence samples (p = 0.02) (Fig 4B). In both Persistence and Clearance samples, relative to cells from healthy donors, PD-1 was significantly upregulated on sE2+ actMBCs (p = 0.04, <0.0001), sE2- actMBCs (p = 0.04, 0.006), sE2+ tlMBC (p = 0.004, <0.0001), and sE2- tlMBC (p = 0.006, 0.006). In both actMBC and tlMBC subsets from Clearance samples, expression of PD-1 was significantly greater on sE2+ cells than sE2- cells (p = 0.006, 0.04) (Fig 4C and 4D). PD-1 expression also was significantly greater on sE2+ actMBC from Clearance vs. Persistence samples (p = 0.0001). We did not observe any statistically significant differences in BTLA, CD22, or CXCR5 expression between sE2+ and sE2- cells in any of the four csBC subsets in either Clearance or Persistence samples. (S6 Fig). Additionally, we did not observe any significant differences in surface marker expression on sE2+ cells in any of the four csBC subsets from pre-Clearance vs. time-matched Persistence samples, early vs. late Persistence samples, or pre- vs. post-Clearance samples (S7–S9 Figs). In summary, FCRL5 was upregulated on sE2+ intMBCs, rMBCs, and actMBCs from both Persistence and Clearance samples. PD-1 was upregulated on both sE2+ and sE2- actMBCs and tlMBC from both Persistence and Clearance samples, with greater expression on sE2+ Clearance actMBCs than sE2+ Persistence actMBCs.

### Correlation of plasma anti-E2 IgG levels with E2-specific B cell phenotypes

**Fig 5:**
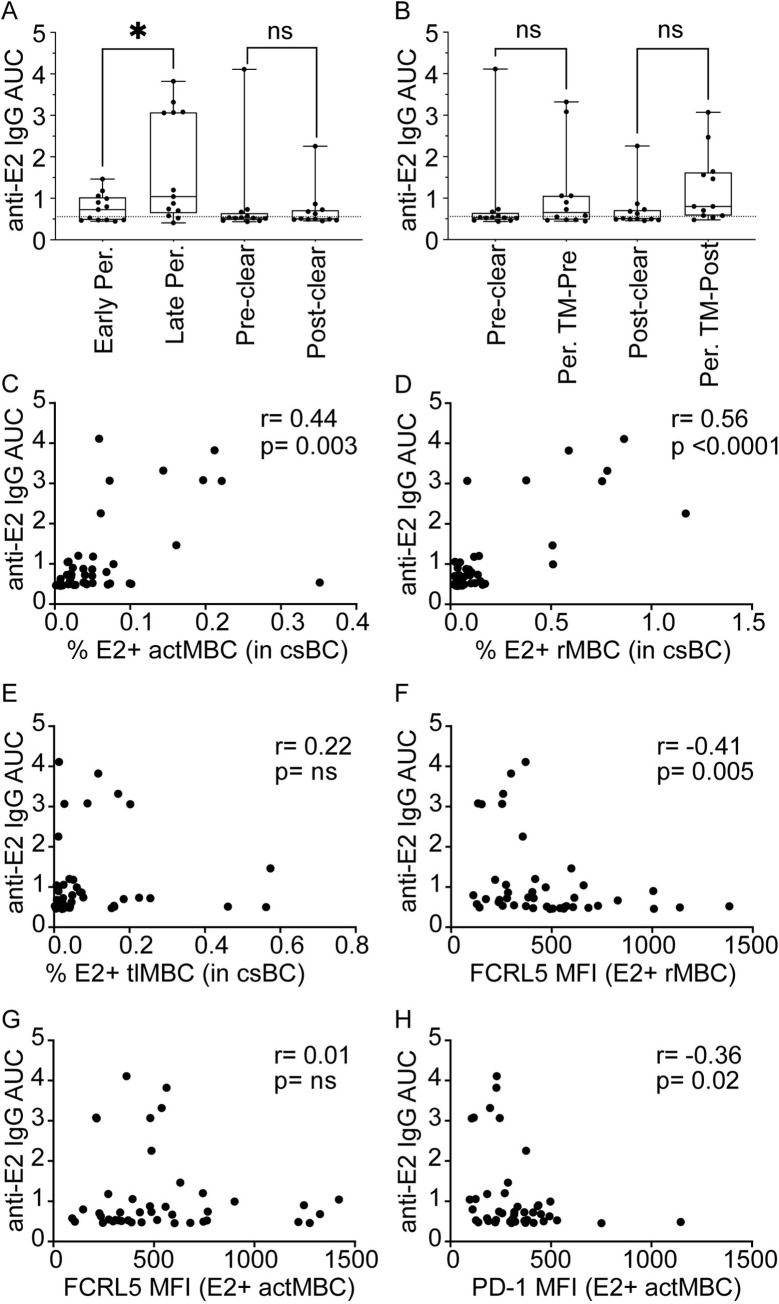
Correlation of plasma anti-E2 IgG levels with E2-specific B cell phenotypes. **(A)** Plasma anti-E2 IgG levels of early or late Persistence and pre- or post-Clearance samples determined by calculating area under the curve (AUC). **(B)** Anti-E2 IgG AUC of Clearance (pre-Clear, post-Clear) or Persistence subjects time-matched with pre- and post-Clearance samples for duration of infection (Per. TM-Pre, Per. TM-Post). **(C)** Correlation of the frequency (%) of sE2+ resting memory B cells (rMBC) among csBC and plasma anti-E2 IgG AUC. **(D)** Correlation of % sE2+ activated MBC (actMBC) among csBC and plasma anti-E2 IgG AUC. **(E)** Correlation of % sE2+ tissue-like memory B cells (tlMBC) among csBC and plasma anti-E2 IgG AUC. **(F)** Correlation of FCRL5 MFI of sE2+ resting MBC (rMBC) and plasma anti-E2 IgG AUC. **(G)** Correlation of FCRL5 MFI of sE2+ actMBC and plasma anti-E2 IgG AUC. **(H)** Correlation of PD-1 MFI of sE2+ actMBC and plasma anti-E2 IgG AUC. For **A** and **B**, horizontal lines indicate means, boxes are inter-quartile range, and whiskers are minimum to maximum. Two-tailed paired Wilcoxon test **(A)** or unpaired Mann-Whitney U test **(B)** for non-normally distributed data was performed with p value adjusted for multiple comparisons using the Bonferroni method. * P ≤ 0.05. For **C-H**, each point indicates a single sample. Early Persistence, late Persistence, pre-Clearance, and post-Clearance samples were included. Correlation r and p values were calculated using the Spearman method.

To evaluate the functional impact *in vivo* of the observed shifts in sE2+ csBC subset frequencies and differences in surface molecule expression, we measured anti-E2 IgG in the plasma of the same blood samples used to evaluate sE2+ B cells. Anti-E2 IgG was quantitated in an ELISA by measuring binding of IgG in serial dilutions of plasma to wells coated with a mixture of the same three sE2 proteins used for B cell staining. Binding area under the curve (AUC) was calculated for each plasma sample (Fig 5A and 5B). The majority of samples had low anti-E2 IgG AUCs, with 6 of 13 early Persistence, 2 of 13 later Persistence, 8 of 12 pre-Clearance samples, and 7 of 12 post-Clearance samples failing to exceed the true-positive cutoff established using negative control normal human plasma. Notably, 17 of 23 (74%) of these anti-E2 IgG negative samples had detectable sE2+ B cells. Also of note, IgG levels varied widely across samples in each study group, with some individuals in each group anti-E2 IgG negative, and some individuals showing relatively high AUC measurements up to 7-times the true positive cutoff value. The highest AUC measurements were taken from samples in the late Persistence and pre-Clearance groups. We observed a small but statistically significant increase in plasma anti-E2 IgG levels from early to later Persistence (p = 0.02), but the differences in anti-E2 IgG AUC between pre- and post-Clearance samples or between pre-Clearance and time-matched Persistence samples were not significant (p = 0.6 and 0.06, respectively) (Fig 5A and 5B). Taken together, these data indicate that anti-E2 IgG levels vary greatly across both Clearance and Persistence subjects, and that many individuals have undetectable plasma anti-E2 IgG levels despite active viremia and detectable sE2+ csBC in circulation.

Since we observed reduced frequency of rMBC and increased frequency of actMBC and tlMBC among sE2+ csBC relative to frequency in sE2- csBC from the same subjects or healthy controls (Fig 3), we assessed the correlation across all HCV+ samples between plasma anti-E2 IgG AUC and the frequencies of sE2+ rMBC, sE2+ actMBC, or sE2+ tlMBC cells among csBC (Fig 5C–5E). We observed a statistically significant positive correlation between plasma anti-E2 IgG AUC and frequency of sE2+ rMBC (r = 0.56, p<0.0001) and with frequency of sE2+ actMBC (r = 0.44, p = 0.003) (Fig 5C and 5D). There was no correlation between IgG AUC and frequency of sE2+ tlMBC (r = 0.22, p = ns) (Fig 5E). Given the positive correlation between IgG AUC and sE2+ rMBC and actMBC frequencies, and the upregulation of FCRL5 on sE2+ cells in these subsets relative to sE2- cells (Fig 4), we next assessed the correlation across all HCV+ samples between IgG AUC and FCRL5 expression on sE2+ rMBC or on sE2+ actMBC (Fig 5F and 5G). We observed a significant negative correlation between IgG AUC and FCRL5 expression on rMBC (r = -0.41, p = 0.005), but no correlation with FCRL5 expression on actMBC (r = 0.01, p = ns). Finally, because we also observed upregulation of PD-1 on sE2+ actMBC (Fig 4), we measured the correlation across all HCV+ samples between IgG AUC and level of PD-1 expression on sE2+ actMBC, demonstrating a significant negative correlation (r = -0.36, p = 0.02) (Fig 5H). Examination of flow plots demonstrated that the increase in FCRL5 or PD-1 MFI in subjects with low anti-E2 IgG titers was driven by increased expression of these molecules across all rMBC or all actMBC, rather than a discreet subset of FCRL5 or PD-1 positive cells (S10 Fig). Taken together, these data indicate that plasma anti-sE2 IgG levels are positively correlated with frequencies of sE2+ rMBC and actMBC, while IgG levels are negatively correlated with levels of FCRL5 expression on sE2+ rMBC and levels of PD-1 expression on sE2+ actMBC.

## Discussion

Approximately 25% of individuals infected with HCV naturally clear the infection, and natural clearance has been associated with early development of high serum titers of anti-E2 IgG [9], and early development of E2-specific bNAbs [10–12, 40]. In chronically infected individuals, anti-E2 bNAbs have been associated with delayed disease progression [7, 41]. However, many HCV-infected individuals, including some people with spontaneous clearance of infection, demonstrate low or delayed anti-E2 IgG responses. Here, we measured plasma anti-E2 IgG levels and used a cocktail of soluble E2 (sE2) proteins to identify sE2+ B cells from longitudinal samples of individuals with a wide range of anti-E2 IgG titers, with either clearance or persistence of HCV infection. We detected circulating, sE2+ csBC in most HCV-infected individuals, including many with low or absent anti-E2 IgG levels. Notably, although the bait antigens used for staining were from genotype 1 viruses, we did not detect a significant difference in sE2+ csBC frequency between genotype 1 and genotype 2 or 3-infected individuals. In sE2+ csBC from both Persistence and Clearance subjects, frequencies of rMBC were reduced, frequencies of actMBC and tlMBC were increased, and expression of FCRL5 was significantly upregulated. We observed a positive correlation between plasma anti-E2 IgG levels and frequencies of sE2+ rMBC and sE2+ actMBC, and a negative correlation between anti-E2 IgG levels and levels of FCRL5 expression on sE2+ rMBC and levels of PD-1 expression on sE2+ actMBC.

In sE2+ csBC of both Persistence and Clearance subjects, we saw reduced frequencies of rMBC and increased frequencies of actMBC and tlMBC. It is interesting that frequencies of sE2+ rMBC and actMBC were each positively correlated with plasma anti-E2 IgG levels, since most plasma antibodies are not produced by these cell types, but rather by antibody secreting cells (ASCs), including plasmablasts and plasma cells. We did not analyze ASCs in this study because they express little or no BCR on their surface, making their antigen specificity difficult to determine directly *ex vivo*. The correlation that we observed between rMBC or actMBC frequencies and antibody titers is consistent with the generally accepted model where, under conditions of antigenic stimulation, rMBC proliferate and differentiate into actMBC, which in turn differentiate into ASCs [42].

Our data indicate that FCRL5 and PD-1 may be important regulators of anti-E2 IgG production *in vivo*. FCRL5, an IgG receptor, is thought to have dual function as either a B cell activator or inhibitor, as it contains both an immunoreceptor tyrosine-based activation motif (ITAM) and two inhibitory motifs (ITIMs) [43, 44]. Many studies of FCRL5 have been done in the context of malaria infection, where high expressions of FCRL5 was seen primarily on tlMBC [31, 32]. In our study, FCRL5 expression was not increased on sE2+ tlMBC. In contrast, FCRL5 expression was upregulated on sE2+ rMBC and actMBC, and levels of expression on rMBC were negatively correlated with plasma anti-E2 IgG levels. This negative correlation between FCRL5 expression on rMBC and IgG levels suggests that FCRL5 may limit proliferation and differentiation of sE2+ rMBC into ASC, thereby reducing anti-E2 IgG production. PD-1 has been extensively studied as a marker of both activation and exhaustion on T cells [29, 45, 46], but its role in B cell function remains elusive [28, 47]. Salimzadeh *et al*. saw upregulation of PD-1 on hepatitis B virus (HBV)-specific B cells during chronic HBV infection, and showed that blockade of PD-1 increased proliferative capacity of HBV-specific B cells *in vitro* [30]. We found that during HCV infection, PD-1 expression was increased on sE2+ actMBC and tlMBC, and the levels of PD-1 expression on actMBC were negatively correlated with anti-E2 IgG levels *in vivo*. While further *in vivo* and *in vitro* studies are needed to validate these observations, these data suggest that PD-1 on actMBC might act in concert with FCRL5 on rMBC in limiting differentiation of these cell types into anti-E2-producing ASCs.

Given a prior study showing an association between early development of high anti-E2 IgG titers and spontaneous clearance of infection [9], it was somewhat surprising that we did not see a difference in plasma anti-E2 IgG levels, FCRL5, or PD-1 expression between the Clearance and Persistence groups, despite testing acute infection samples and matching the two groups for duration of infection. This discrepancy with prior studies could be the result of differences in E2 strains used for testing or in timing of samples used for analysis. Alternatively, given the relatively small number of subjects studied, and the heterogeneity in anti-HCV antibody responses in each group, we may not have had adequate power to detect differences between the groups. Since anti-E2 IgG levels did not differ significantly between Persistence and Clearance groups, it is not surprising that we also did not see differences in sE2+ FCRL5 or PD-1 expression, since IgG levels were negatively correlated with expression of these molecules on rMBC or actMBC. Nevertheless, our findings here of mechanisms potentially controlling levels of anti-E2 antibody expression in both Persistence and Clearance subjects are highly relevant for vaccine development, since a successful vaccine will likely need to induce high titers of anti-E2 IgG.

In summary, using a rationally selected cocktail of sE2 proteins, we detected E2-specific csBC in acute infection samples from the majority of Persistence and Clearance subjects, including many individuals with low or undetectable plasma anti-E2 IgG levels. In the sE2+ csBC compartment, frequencies of resting memory B cells (rMBC) were reduced, frequencies of activated MBC (actMBC) and tissue-like-memory B cells (tlMBC) were increased, and expression of FCRL5 was significantly upregulated. Plasma anti-E2 IgG levels were positively correlated with frequencies of sE2+ rMBC and sE2+ actMBC. Levels of FCRL5 expression on sE2+ rMBC and levels of PD-1 expression on sE2+ actMBC were negatively correlated with plasma anti-E2 IgG levels. Our findings show that B cell dysfunction begins in acute HCV infection, and overexpression of FCRL5 on rMBC and PD-1 on actMBC is associated with reduced anti-E2 IgG production *in vivo*. Further studies are warranted to elucidate whether limiting the upregulation of these molecules could potentially generate higher titers of protective antibodies against HCV or other pathogens.

## Materials and methods

### Ethics statement

This research was approved by the Johns Hopkins University School of Medicine’s Institutional Review Board (IRB). Prior to blood collection, all participants provided informed written consent.

### Study subjects

PBMC and plasma samples were obtained from the Baltimore Before and After Acute Study of Hepatitis (BBAASH) cohort [48], a prospective cohort of injection drug users in Baltimore who are followed from before the time they are infected with HCV, through spontaneous clearance/persistence of HCV.

### HCV viral load and subject groups

HCV viral loads (IU/mL) were quantified after RNA extraction with the use of commercial real-time reagents (Abbot HCV Real-time Assay) migrated onto a research-based real-time PCR platform (Roche 480 LightCycler). HCV seropositivity was determined using the Ortho HCV version 3.0 ELISA Test System (Ortho Clinical Diagnostics). Based on infection outcome, all subjects were assigned to either Clearance or Persistence groups. Clearance was defined as undetectable HCV RNA for a period of at least 60 days in individuals with detectable anti-HCV antibodies. Persistence was defined as persistent HCV RNA viremia for more than 1 year. Day 0 of infection was estimated as the midpoint date of the last negative and first positive HCV RNA test. Days post infection (dpi) was calculated as that timepoint’s date minus day 0’s date.

### Generation and selection of soluble E2 (sE2) strains for B cell staining

Genes encoding E2 ectodomains, residues 384–643, of 89 distinct genotype 1 HCV strains were cloned from a previously described library of E1E2 clones [34] into a mammalian expression vector. sE2 expression and ELISA was performed as previously described [10]. Reverted unmutated ancestor (rua) variants of each mAb were inferred with IMGT/V-QUEST [49, 50] using complete sequences of heavy- and light-chain variable domains and generated by site-directed mutagenesis of mature mAb plasmids (Quikchange, Promega). Binding OD_450_ was normalized for relative protein concentration of each sE2 strain using OD_450_ of anti-HIS binding for each strain. sE2 strains were grouped by hierarchical clustering based on these normalized OD_450_ values across 24 mAbs, using Ward’s minimum variance method in the hclust R package. An unrooted clustering tree was created with the ape R library, as previously described [51]. For extended description, see Supplemental Methods (S1 Text).

### Cell staining and flow cytometry

PBMCs were isolated from blood using a Ficoll density separation gradient. Anti-CD81 antibody (BD Cat #555675) at 5 μg/mL and Fc blocker (BD Cat #564220) diluted in FACS Buffer (1x PBS with 1% BSA) was added to the cells and incubated for 30 minutes at 4°C. sE2 protein was added to the cells at 5 μg/mL and incubated at room temperature for 30 min. Conjugated antibodies (S1 Table) and viability stain were added to the cells and incubated for an additional 30 min. The cells were washed two or three more times before running the cells on BD Biosciences LSR II instrument for FlowJo analysis or sorting populations of interest using the MoFlo (BD) for culture experiments. For extended description, see Supplemental Methods (S1 Text).

### Sorted E2 specific and nonspecific B cells and cultures

20 x 10^6^ PBMCs were stained with his tagged cocktail antigens (1a157, 1b09, and 1b21) along with propidium iodide (PI), CD10-PE, CD19-BV421, CD3-APC H7, IgM-BB515, IgD-BB515, and Anti His-A647. Sorted B cells were cultured *in vitro* using previously published methods [39]. Briefly, the B cells were sorted into culture medium and seeded in various densities in clear u-bottom 96-well cell culture plates along with irradiated mouse 3T3 fibroblast cells expressing CD40L, IL-2 (Fisher Scientific Cat #202IL050CF), and IL-21 (Fisher Scientific Cat #8879IL050). 3T3-CD40L cells were obtained through the NIH AIDS Reagent Program, Division of AIDS, NIAID, NIH: Cat #12535 3T3-msCD40L cells, from Dr. Mark Connors. The plated cells then were incubated at 37°C in 5% CO_2_ and on the 14^th^ day, supernatants were collected for use in ELISAs for antibody production.

### ELISA to detect E2-specific IgG in B cell supernatants or plasma

ELISAs were preformed as previously described [10]. Immulon 2b microtiter plates were coated with lectin and then a mixture of 1a157, 1b09, and 1b21 sE2 protein at a total concentration of 1 μg/mL. Culture supernatants were then added undiluted. Plasma was diluted 5-fold from 1:100 to 1:4.88E+09. Pre-immune plasma (PIP) or culture media were used to set the true positive cutoff threshold. Area under the curve (AUC) was calculated using Prism (Graphpad software). For extended description, see Supplemental Methods (S1 Text).

### Statistical analysis

FlowJo software was used to analyze the flow cytometry results. Statistical analyses were performed in Prism (Graphpad software). Two-group comparisons were performed with t tests if data were normally distributed based on the Shapiro Wilk normality test or the Mann Whitney rank test if data were not normally distributed, matched only in comparisons of pre- to post-clear and early to late persistence. Multi-group comparisons were performed using one-way ANOVA if data were normally distributed based on the Shapiro Wilk normality test or the Kruskal-Wallis test if data were not normally distributed, with p values adjusted for multiple comparisons using the Benjamini, Krieger and Yekutieli method.

## Supporting information

S1 Text Supplemental Methods. (DOCX)

S1 Fig Binding of 12 mature E2-specific mAbs and their reverted unmutated ancestor (rua) mAbs to 89 distinct genotype 1 sE2 proteins. (TIF)

S2 Fig Gating schematic of sE2-specific (sE2+) or sE2-nonspecific (sE2-) csBC. (TIF)

S3 Fig sE2 binding of cultured sE2-specific (sE2+) and nonspecific (sE2-) csBC supernatants. (TIF)

S4 Fig Equivalent frequencies of MBC subsets among sE2-specific (sE2+) csBC from different groups. (TIF)

S5 Fig Equivalent expression of BTLA, CD22, CXCR5, FCRL5, and PD-1 on sE2-specific (sE2+) csBC from different groups. (TIF)

S6 Fig Equivalent expression of BTLA, CD22, and CXCR5 on sE2-nonspecific (sE2-) or sE2-specific (sE2+) csBC subsets. (TIF)

S7 Fig Equivalent expression of BTLA, CD22, CXCR5, FCRL5, and PD-1 on sE2-specific (sE2+) csBC subsets from pre-Clearance and time-matched Persistence samples. (TIF)

S8 Fig Equivalent expression of BTLA, CD22, CXCR5, FCRL5, and PD-1 on sE2-specific (sE2+) csBC subsets among pre- or post- Clearance samples. (TIF)

S9 Fig Equivalent expression of BTLA, CD22, CXCR5, FCRL5, and PD-1 on sE2-specific (sE2+) csBC subsets among early or late Persistence samples. (TIF)

S10 Fig Flow plots of FCRL5 or PD-1 expression on rMBC or actMBC. (TIF)

S1 Table Flow cytometry reagents used. (TIF)
