# Supplementary material for: B cell overexpression of FCRL5 and PD-1 is associated with low antibody titers in HCV infection
Source: PLoS Pathog. 2022 Jan 6;18(1):e1010179. doi: 10.1371/journal.ppat.1010179 (PMC8769295; doi:10.1371/journal.ppat.1010179)
Supplement: S1 Text — (DOCX) [file ppat.1010179.s001.docx]

**Supplemental Methods**

**Generation and selection of soluble E2 (sE2) strains for B cell staining**

Genes encoding E2 ectodomains, residues 384-645, of 89 distinct genotype 1 HCV strains were cloned from a previously described library of E1E2 clones [1] into a mammalian expression vector (phCMV3-Ig Kappa-HIS, a gift of Leopold Kong, The Scripps Research Institute, La Jolla, California, USA). The vector allows expression of soluble E2 protein with a C-terminal His tag and an N-terminal murine Ig kappa chain leader signal for efficient protein secretion. sE2 expression and ELISA was performed as previously described [2]. Each sE2 construct was co-transfected with pAdvantage plasmid (Promega) into HEK293T cells and incubated for 72 hours at 37°C. sE2-containing supernatant were diluted 1:10 and used to coat ELISA plates. Twelve previously described E2-specific mAbs [3, 4] (Supplemental Figure 1) were synthesized (IDT) and cloned into the pTT5-based vectors (NRC Biotechnology Research Institute). Reverted unmutated ancestor (rua) variants of each mAb were inferred with IMGT/V-QUEST [5, 6] using complete sequences of heavy- and light-chain variable domains and generated by site-directed mutagenesis of mature mAb plasmids (Quikchange, Promega). IgGs were produced in HEK293-6E cells by co-transfecting with appropriate heavy- and light-chain plasmids. HiTrap Protein A HP columns (GE Healthcare) were used to isolate IgGs from filtered culture supernatants followed by purification by size exclusion chromatography (SEC). Binding of mature mAbs, rua mAbs, or mouse monoclonal anti-6x His-tag antibody (Thermo Fisher Scientific) to each sE2 was measured at 10 µg/mL mAb concentration. Binding OD_450_ was normalized for relative protein concentration of each sE2 strain using OD_450_ of anti-HIS binding for each strain. sE2 strains were grouped by hierarchical clustering based on these normalized OD_450_ values across 24 mAbs, using Ward’s minimum variance method in the hclust R package. An unrooted clustering tree was created with the ape R library, as previously described [7].

**Cell staining and flow cytometry**

PBMCs were isolated from blood using a Ficoll density separation gradient. Anti-CD81 antibody (BD Cat #555675) at 5 µg/mL and Fc blocker (BD Cat #564220) diluted in FACS Buffer (1x PBS with 1% BSA) was added to the cells and incubated for 30 minutes on ice or 4°C. A CD81 blocking step was included to minimize non-specific binding of E2 to lymphocytes, since CD81 is an HCV receptor that is expressed ubiquitously by lymphocytes [8]. sE2 protein was added to the cells at 5 µg/mL and incubated at room temperature for 30 min. Conjugated antibodies (S1 Table) and viability stain were added to the cells and incubated for an additional 30 min. The cells were washed two or three more times before running the cells on BD Biosciences LSR II instrument for FlowJo analysis or sorting populations of interest using the MoFlo (BD) for culture experiments.

**ELISA to detect antibody production from B cell cultures**

To detect human IgG in culture supernatants, we followed an ELISA protocol that was shared by Dr. Steven Foung (Stanford University School of Medicine). Briefly, 96-well Maxisorp plates were coated with capture antibody of Jackson Alkaline Phosphatase AffiniPure Goat Anti-Human IgG, Fcγ fragment specific (Fisher Scientific Cat #109-055-098). Culture supernatants or standards were added and incubated on a rocking platform. Goat Anti-Human IgG Fab Secondary Antibody (MyBioSource Cat #MBS8216436) was used at a dilution of 1:4,000. Finally, alkaline phosphatase yellow (pNPP) liquid substrate system was added, and absorbance read at lambda 405 and lambda 570. To detect production of E2-specific IgG, ELISAs were performed as previously described [2]. Immulon 2b microtiter plates were coated with lectin followed by incubation with sE2 proteins (50 µL at 1 µg/mL) overnight. The next day, the plates were blocked with PBS-TMG (PBS + 0.5% tween 20+ 1% non-fat dry milk+1% goat serum) and then incubated with B cell culture supernatants or standards. Anti-human IgG-HRP (BD-Pharmingen Cat #555788) was used at a 1:4,000 dilution and finally adding TMB peroxidase substrate to develop and 1N sulfuric acid to stop the reaction and reading the plates at absorbance of 450 nm.

Supplemental Methods References

1. El-Diwany R, Cohen VJ, Mankowski MC, Wasilewski LN, Brady JK, Snider AE, et al. Extra-epitopic hepatitis C virus polymorphisms confer resistance to broadly neutralizing antibodies by modulating binding to scavenger receptor B1. PLoS Pathog. 2017;13(2):e1006235. doi: 10.1371/journal.ppat.1006235. PubMed PMID: 28235087; PubMed Central PMCID: PMCPMC5342271.

2. Kinchen VJ, Zahid MN, Flyak AI, Soliman MG, Learn GH, Wang S, et al. Broadly Neutralizing Antibody Mediated Clearance of Human Hepatitis C Virus Infection. Cell Host Microbe. 2018;24(5):717-30 e5. doi: 10.1016/j.chom.2018.10.012. PubMed PMID: 30439341; PubMed Central PMCID: PMCPMC6250073.

3. Bailey JR, Flyak AI, Cohen VJ, Li H, Wasilewski LN, Snider AE, et al. Broadly neutralizing antibodies with few somatic mutations and hepatitis C virus clearance. JCI Insight. 2017;2(9). doi: 10.1172/jci.insight.92872. PubMed PMID: 28469084; PubMed Central PMCID: PMCPMC5414559.

4. Colbert MD, Flyak AI, Ogega CO, Kinchen VJ, Massaccesi G, Hernandez M, et al. Broadly Neutralizing Antibodies Targeting New Sites of Vulnerability in Hepatitis C Virus E1E2. J Virol. 2019;93(14). doi: 10.1128/JVI.02070-18. PubMed PMID: 31068427; PubMed Central PMCID: PMCPMC6600205.

5. Brochet X, Lefranc M-P, Giudicelli V. IMGT/V-QUEST: the highly customized and integrated system for IG and TR standardized V-J and V-D-J sequence analysis. Nucleic Acids Research. 2008;36(suppl_2):W503-W8. doi: 10.1093/nar/gkn316.

6. Giudicelli V, Brochet X, Lefranc MP. IMGT/V-QUEST: IMGT standardized analysis of the immunoglobulin (IG) and T cell receptor (TR) nucleotide sequences. Cold Spring Harb Protoc. 2011;2011(6):695-715. doi: 10.1101/pdb.prot5633. PubMed PMID: 21632778.

7. Pierce BG, Keck ZY, Lau P, Fauvelle C, Gowthaman R, Baumert TF, et al. Global mapping of antibody recognition of the hepatitis C virus E2 glycoprotein: Implications for vaccine design. Proc Natl Acad Sci U S A. 2016. doi: 10.1073/pnas.1614942113. PubMed PMID: 27791171; PubMed Central PMCID: PMCPMC5111724.

8. Cherukuri A, Shoham T, Sohn HW, Levy S, Brooks S, Carter R, et al. The Tetraspanin CD81 Is Necessary for Partitioning of Coligated CD19/CD21-B Cell Antigen Receptor Complexes into Signaling-Active Lipid Rafts. The Journal of Immunology. 2004;172(1):370-80. doi: 10.4049/jimmunol.172.1.370.
