# Supplementary figures and images for: B cell overexpression of FCRL5 and PD-1 is associated with low antibody titers in HCV infection
Source: PLoS Pathog. 2022 Jan 6;18(1):e1010179. doi: 10.1371/journal.ppat.1010179 (PMC8769295; doi:10.1371/journal.ppat.1010179)

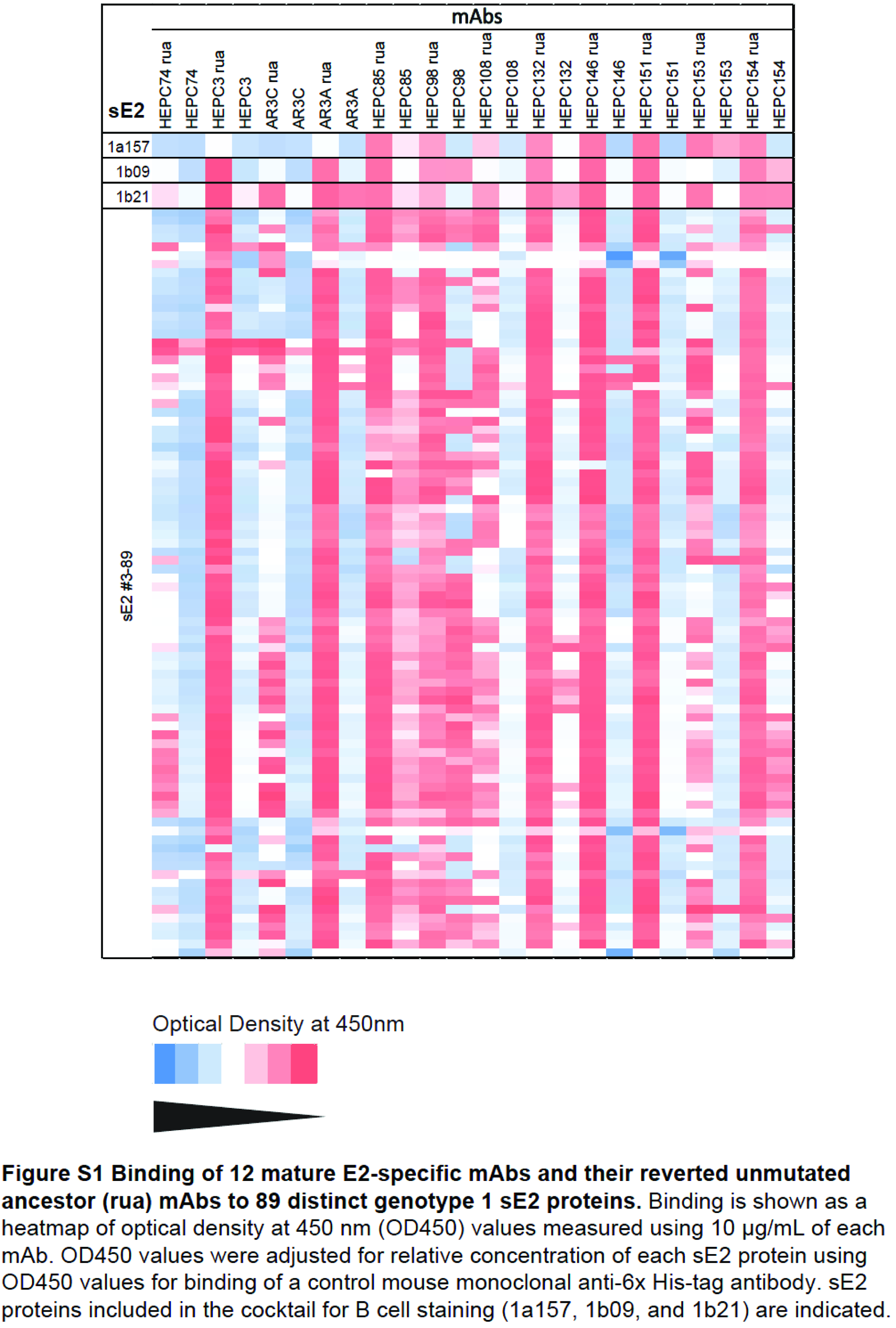

Supplement: S1 Fig — (TIF) [file ppat.1010179.s002.tif]

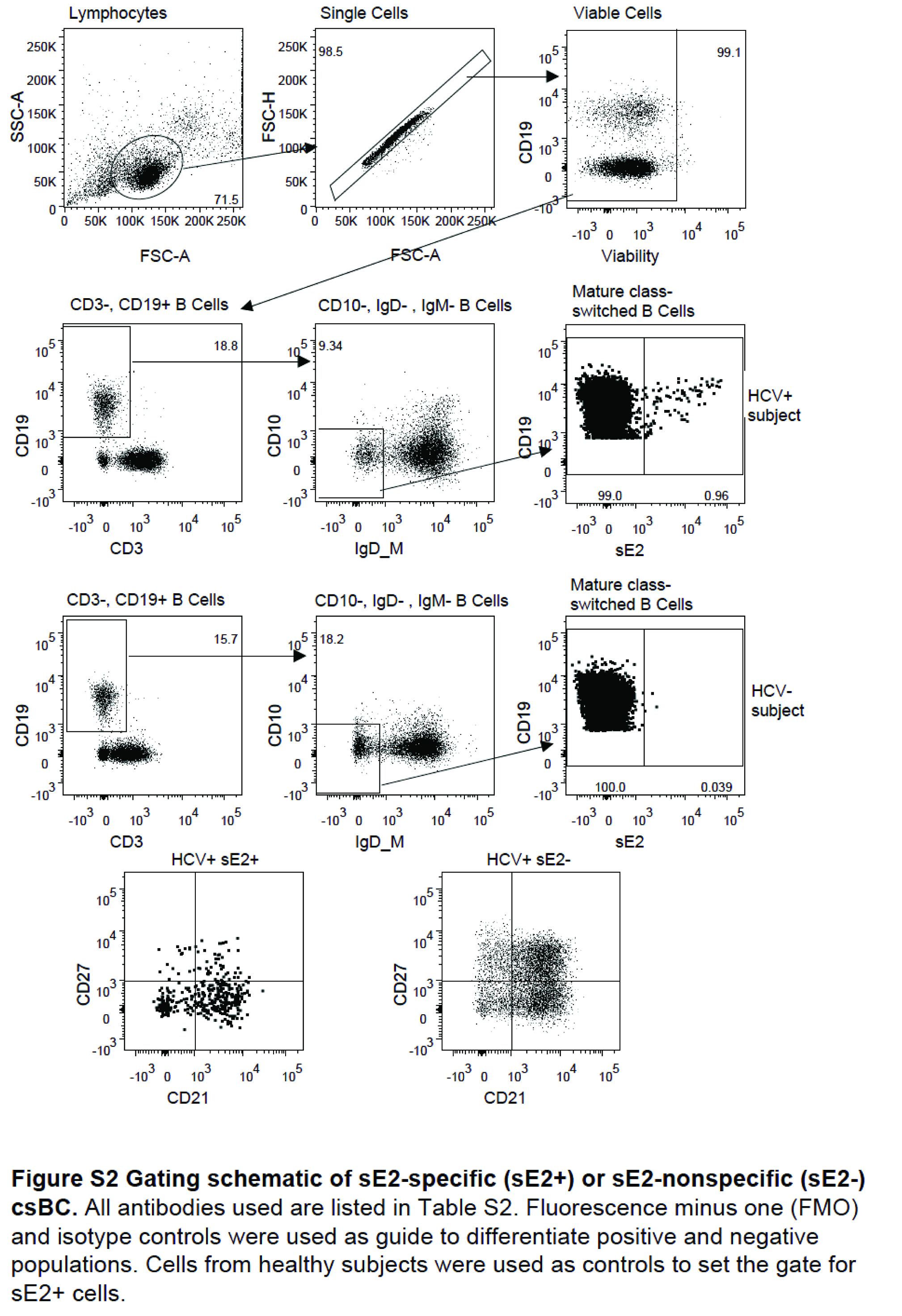

Supplement: S2 Fig — (TIF) [file ppat.1010179.s003.tif]

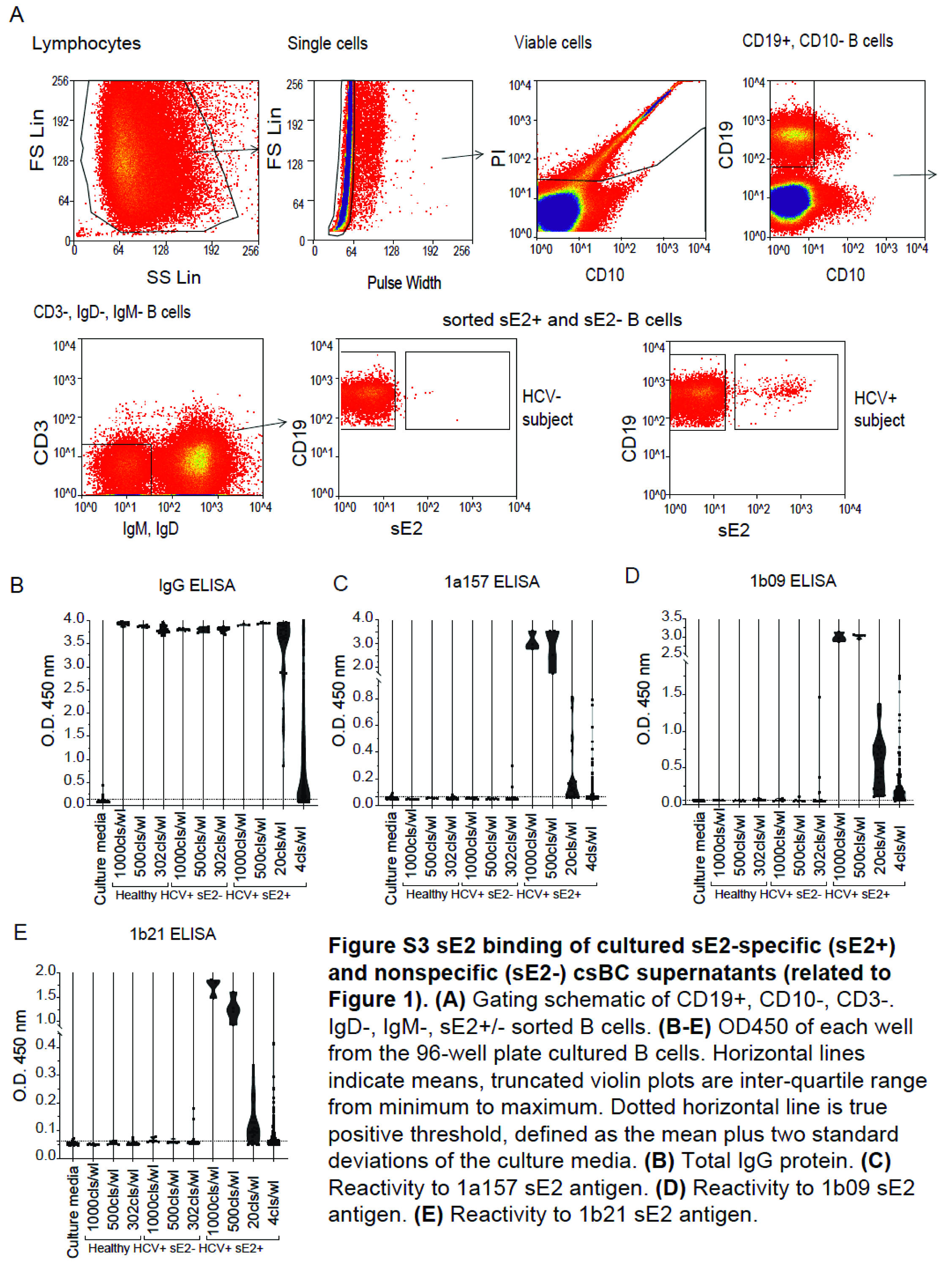

Supplement: S3 Fig — (TIF) [file ppat.1010179.s004.tif]

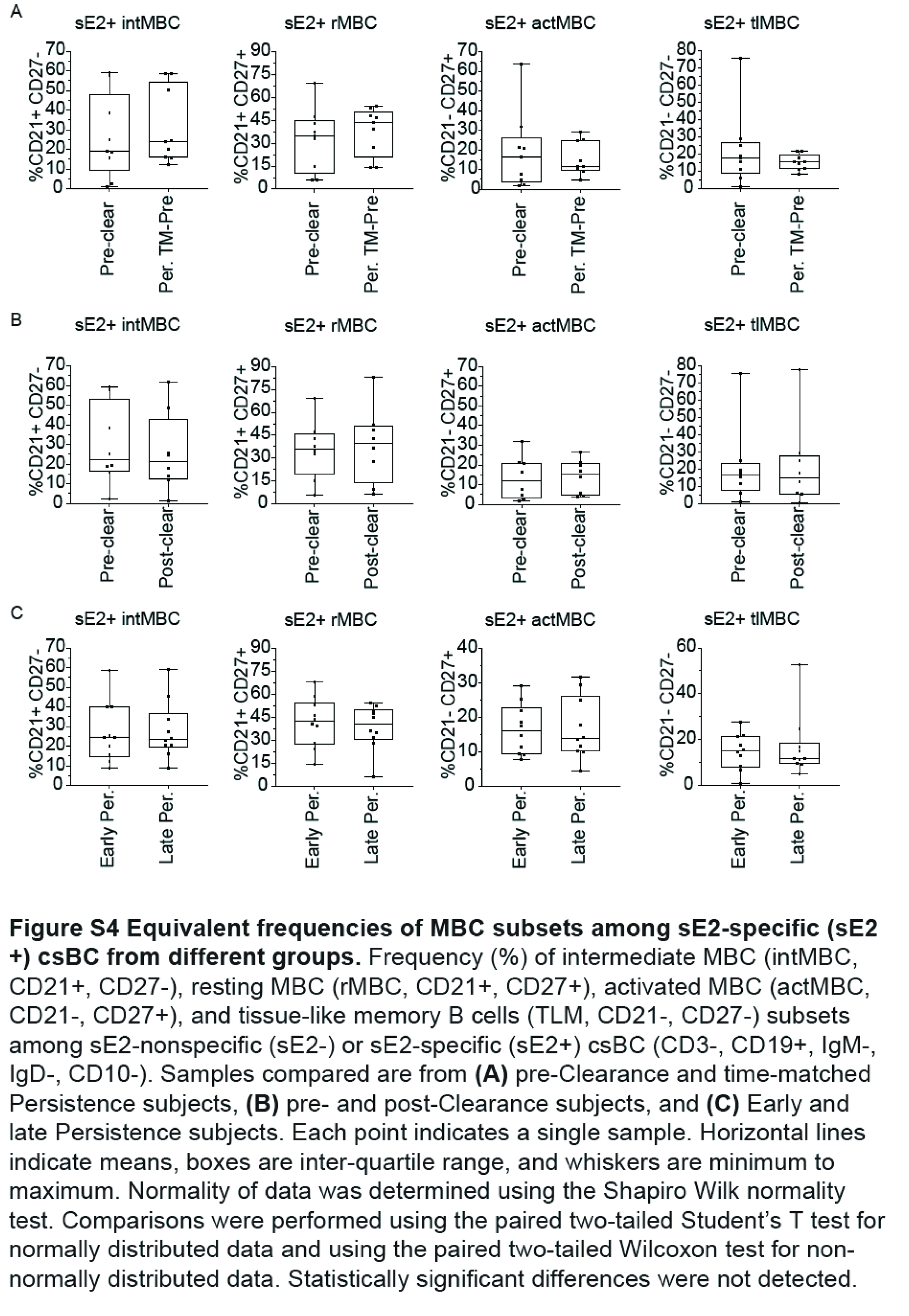

Supplement: S4 Fig — (TIF) [file ppat.1010179.s005.tif]

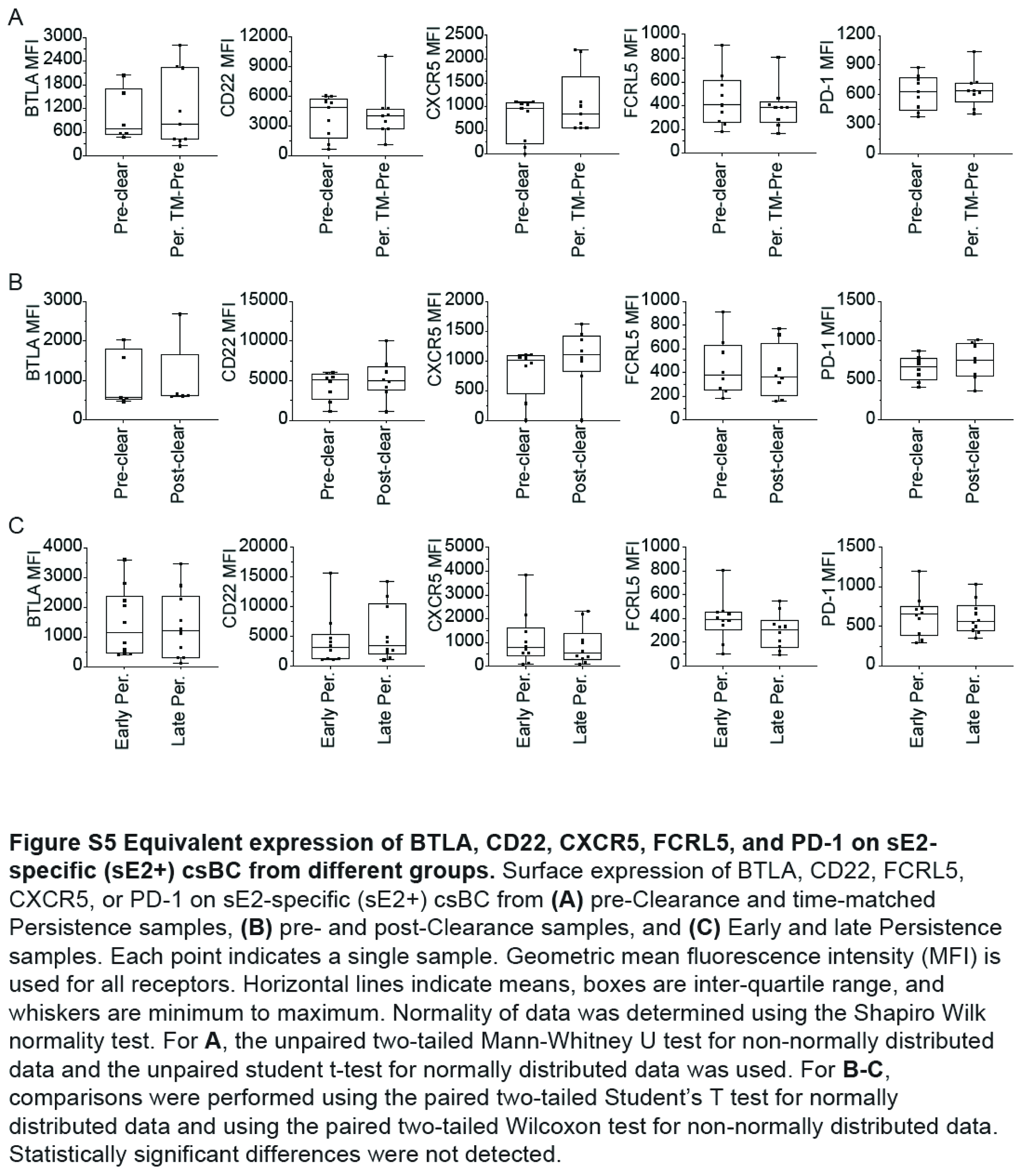

Supplement: S5 Fig — (TIF) [file ppat.1010179.s006.tif]

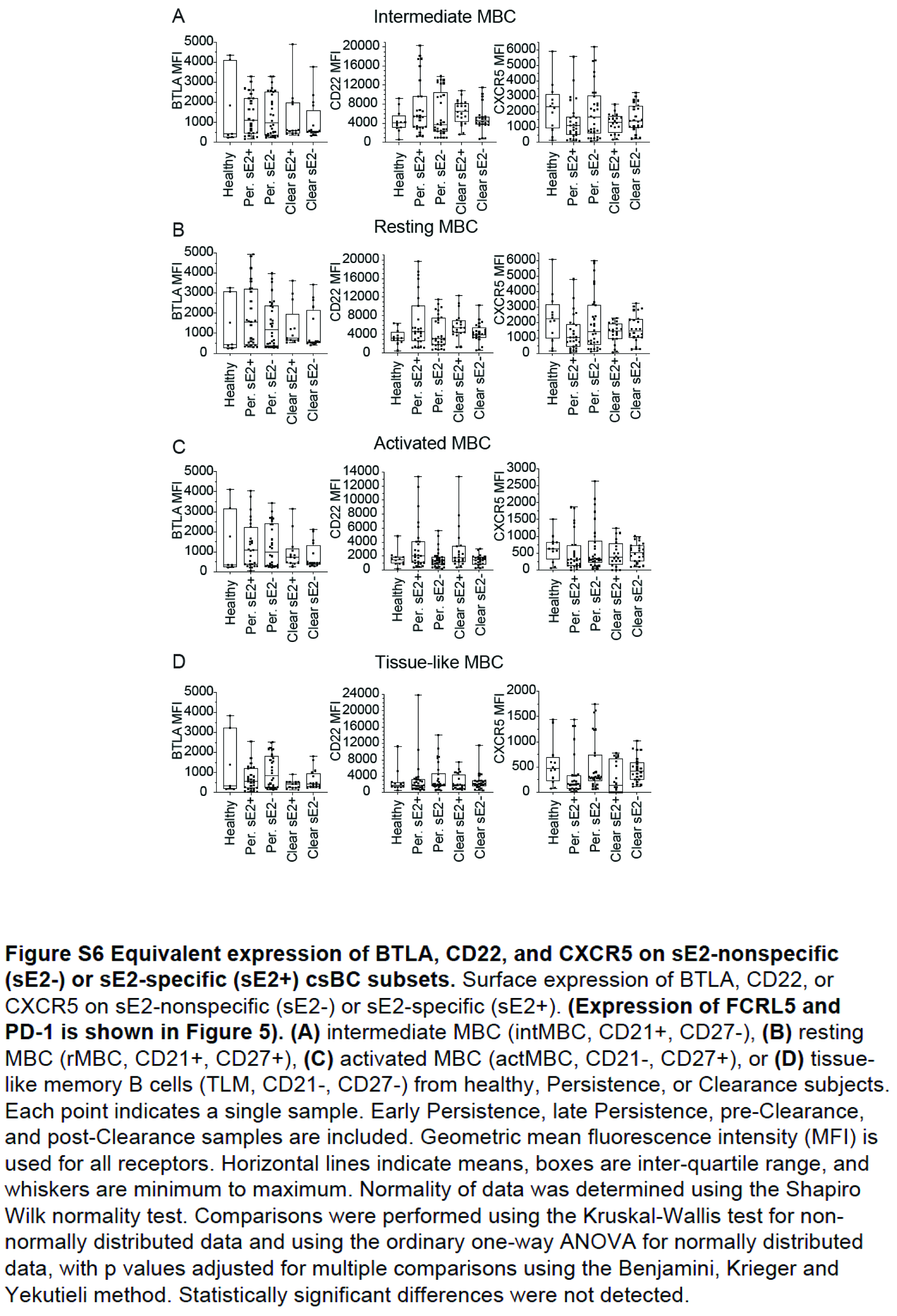

Supplement: S6 Fig — (TIF) [file ppat.1010179.s007.tif]

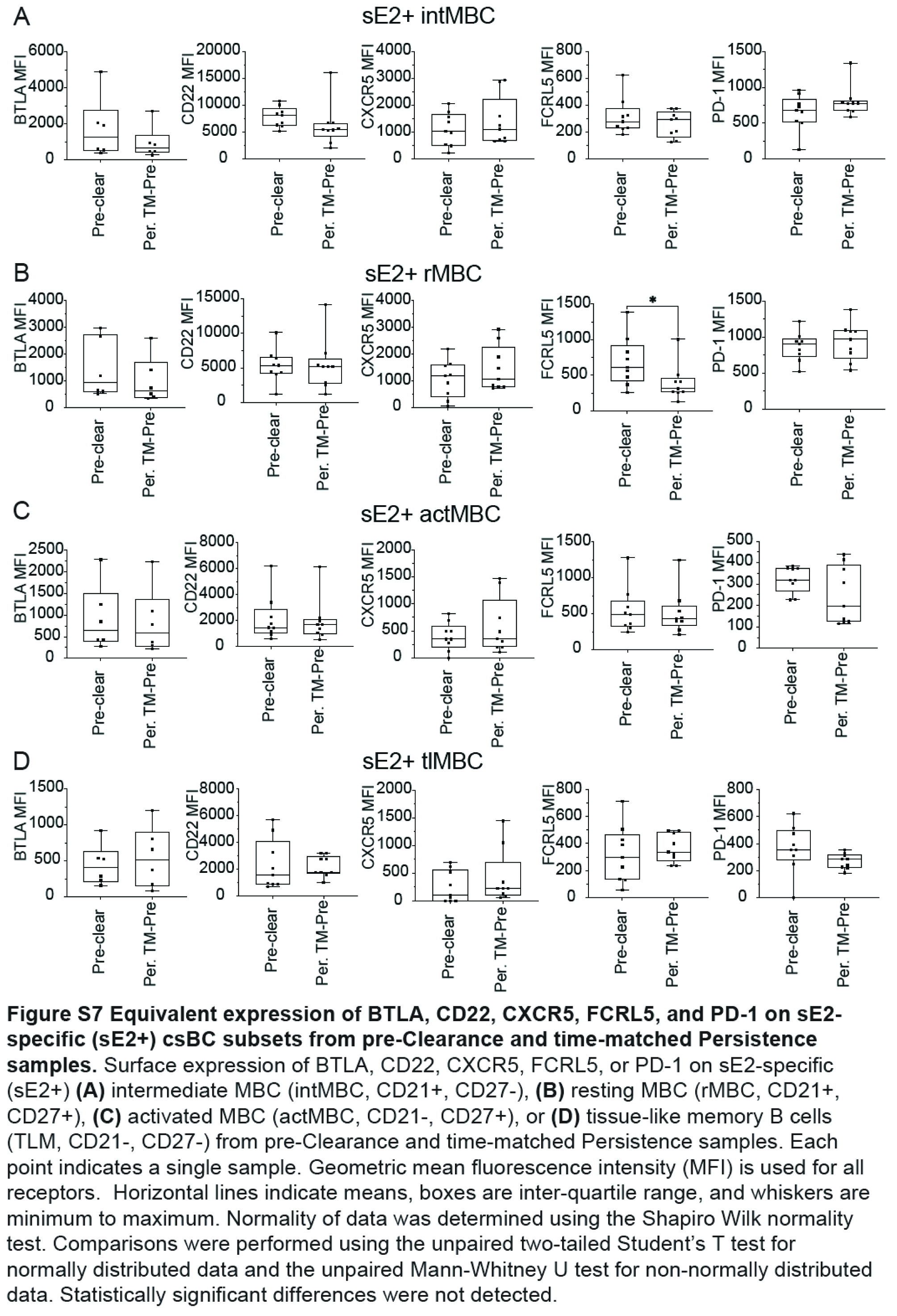

Supplement: S7 Fig — (TIF) [file ppat.1010179.s008.tif]

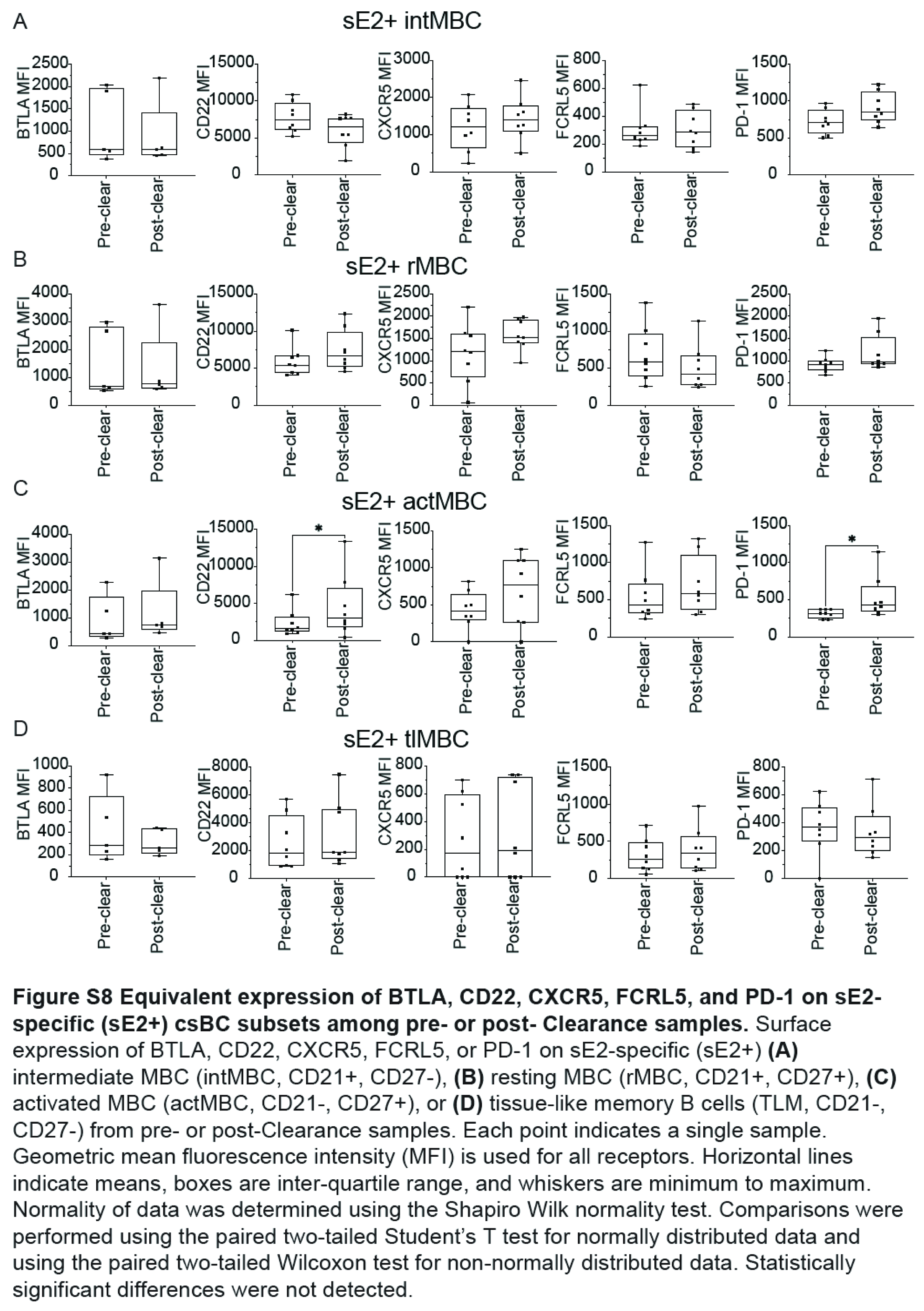

Supplement: S8 Fig — (TIF) [file ppat.1010179.s009.tif]

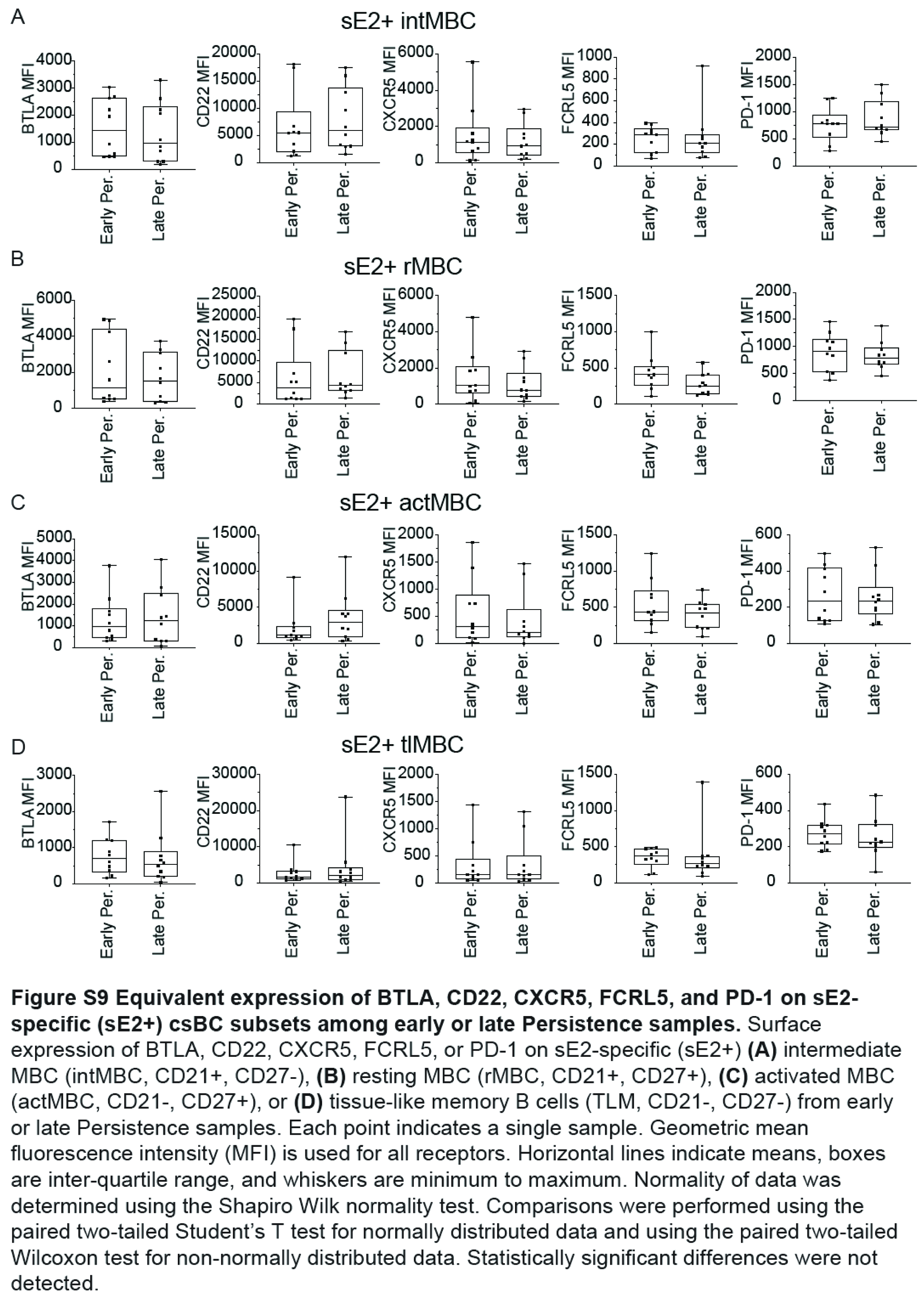

Supplement: S9 Fig — (TIF) [file ppat.1010179.s010.tif]

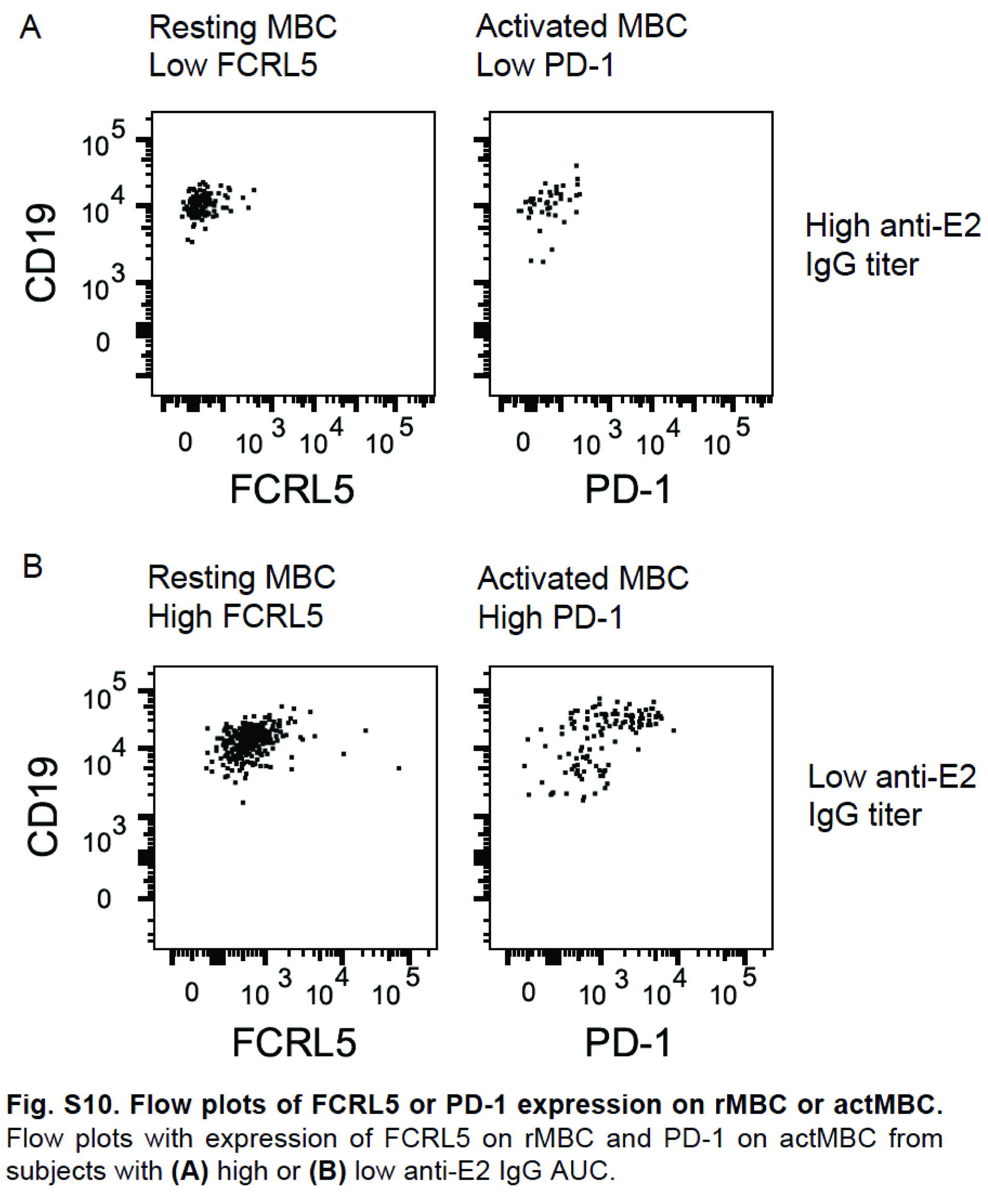

Supplement: S10 Fig — (TIF) [file ppat.1010179.s011.tif]

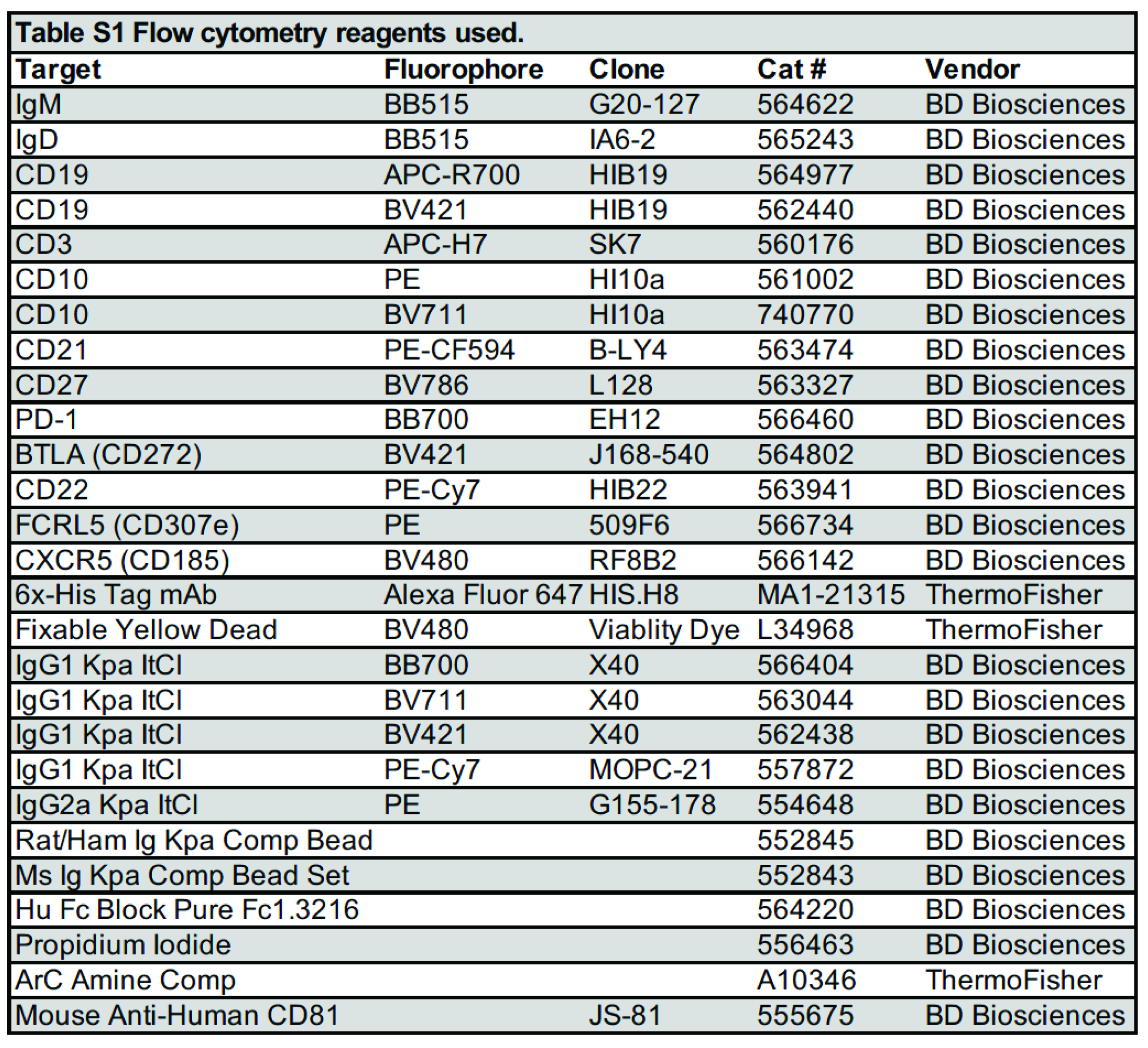

Supplement: S1 Table — (TIF) [file ppat.1010179.s012.tif]
